# Supplementary material for: Single cell cortical bone transcriptomics define novel osteolineage gene sets altered in chronic kidney disease
Source: Front Endocrinol (Lausanne). 2023 Jan 26;14:1063083. doi: 10.3389/fendo.2023.1063083 (PMC9910177; doi:10.3389/fendo.2023.1063083)
Supplement: Supplementary file 1 [file DataSheet_1.pdf]

gene	p_val	avg_log2FC	pct.1	pct.2	p_val_adj	cluster
Serpine2	5.99E-64	3.43216923	0.96	0.305	3.32E-59	Tnc_Mmp13_hiX
Tnc	1.09E-62	3.88254704	0.855	0.189	6.05E-58	Tnc_Mmp13_hiX
Cdh11	6.52E-57	2.57482768	0.766	0.243	3.61E-52	Tnc_Mmp13_hiX
Lifr	1.73E-47	2.84253636	0.798	0.421	9.60E-43	Tnc_Mmp13_hiX
Pdgfrb	6.11E-47	2.53300266	0.589	0.054	3.39E-42	Tnc_Mmp13_hiX
Olfml2b	4.93E-42	2.51145774	0.669	0.137	2.73E-37	Tnc_Mmp13_hiX
Mmp13	3.19E-41	4.32070398	0.669	0.092	1.77E-36	Tnc_Mmp13_hiX
Postn	1.53E-39	2.70088708	0.637	0.109	8.47E-35	Tnc_Mmp13_hiX
Cfh	6.34E-39	2.28695593	0.895	0.364	3.51E-34	Tnc_Mmp13_hiX
Islr	1.26E-38	2.32787752	0.565	0.106	6.98E-34	Tnc_Mmp13_hiX
Cp	4.73E-38	2.17747829	0.661	0.132	2.62E-33	Tnc_Mmp13_hiX
Loxl1	7.14E-37	2.2017403	0.5	0.043	3.96E-32	Tnc_Mmp13_hiX
Gdpd2	6.16E-36	2.57165711	0.435	0.009	3.41E-31	Tnc_Mmp13_hiX
Adamts2	1.40E-35	1.82414547	0.734	0.3	7.77E-31	Tnc_Mmp13_hiX
Rn18s	5.09E-35	2.18166234	1	0.998	2.82E-30	Tnc_Mmp13_hiX
Zfhx4	1.75E-34	1.83057533	0.589	0.121	9.72E-30	Tnc_Mmp13_hiX
Col12a1	4.49E-34	2.36832337	0.637	0.118	2.49E-29	Tnc_Mmp13_hiX
Dpysl3	6.49E-34	1.78302644	0.645	0.248	3.59E-29	Tnc_Mmp13_hiX
Spp1	9.88E-34	3.10306069	0.79	0.364	5.47E-29	Tnc_Mmp13_hiX
Olfml3	1.17E-33	2.0129727	0.758	0.27	6.51E-29	Tnc_Mmp13_hiX
Tpm1	1.38E-33	1.9867228	0.871	0.489	7.66E-29	Tnc_Mmp13_hiX
Fgfr2	5.58E-33	1.69599742	0.589	0.113	3.09E-28	Tnc_Mmp13_hiX
Colec12	1.13E-32	1.83612001	0.645	0.199	6.25E-28	Tnc_Mmp13_hiX
Col6a1	1.36E-32	2.40576984	0.677	0.156	7.52E-28	Tnc_Mmp13_hiX
Serping1	8.04E-32	2.97943161	0.548	0.102	4.46E-27	Tnc_Mmp13_hiX
Sdc2	1.51E-31	1.94533061	0.637	0.206	8.39E-27	Tnc_Mmp13_hiX
Fap	1.57E-31	2.04026232	0.782	0.296	8.71E-27	Tnc_Mmp13_hiX
Antxr1	3.03E-31	1.79791964	0.565	0.123	1.68E-26	Tnc_Mmp13_hiX
Ogn	3.88E-31	1.98890645	0.379	0.012	2.15E-26	Tnc_Mmp13_hiX
Vdr	1.99E-30	1.95113576	0.532	0.18	1.10E-25	Tnc_Mmp13_hiX
Slit2	2.95E-30	1.86325948	0.548	0.154	1.64E-25	Tnc_Mmp13_hiX
Cst3	1.11E-29	1.90663998	0.96	0.851	6.17E-25	Tnc_Mmp13_hiX
Igfbp5	1.96E-29	3.59310498	0.589	0.109	1.09E-24	Tnc_Mmp13_hiX
Neo1	7.65E-29	1.58396507	0.556	0.177	4.24E-24	Tnc_Mmp13_hiX
Foxc1	1.04E-28	1.82996573	0.589	0.163	5.78E-24	Tnc_Mmp13_hiX
Col6a2	2.25E-28	2.23821761	0.629	0.139	1.25E-23	Tnc_Mmp13_hiX
Lrp1	3.33E-28	1.58237208	0.726	0.324	1.84E-23	Tnc_Mmp13_hiX
Ptprd	1.12E-27	1.78626076	0.702	0.255	6.18E-23	Tnc_Mmp13_hiX
Ccdc80	1.23E-27	1.84444414	0.742	0.305	6.79E-23	Tnc_Mmp13_hiX
Fbn1	1.85E-27	1.95251404	0.435	0.047	1.03E-22	Tnc_Mmp13_hiX
Bicc1	1.39E-26	1.68472349	0.589	0.206	7.69E-22	Tnc_Mmp13_hiX
Cpxm1	3.05E-26	1.72407846	0.419	0.052	1.69E-21	Tnc_Mmp13_hiX
Mxra8	3.26E-26	1.58028432	0.726	0.359	1.81E-21	Tnc_Mmp13_hiX
Fstl1	3.79E-26	2.08026293	0.597	0.243	2.10E-21	Tnc_Mmp13_hiX
S1pr3	4.61E-26	1.69822353	0.484	0.156	2.56E-21	Tnc_Mmp13_hiX

Lrp4	6.03E-26	1.77357143	0.621	0.31	3.34E-21	Tnc_Mmp13_hiX
Gas1	1.48E-25	1.6477645	0.476	0.087	8.18E-21	Tnc_Mmp13_hiX
Prrx1	3.11E-25	1.50455068	0.54	0.137	1.72E-20	Tnc_Mmp13_hiX
Lum	3.92E-25	2.35749417	0.71	0.293	2.17E-20	Tnc_Mmp13_hiX
Col8a1	9.44E-25	2.2992036	0.387	0.033	5.23E-20	Tnc_Mmp13_hiX
Tbx2	4.06E-24	1.64756858	0.532	0.173	2.25E-19	Tnc_Mmp13_hiX
Cxcl14	4.42E-24	2.16667734	0.323	0.009	2.45E-19	Tnc_Mmp13_hiX
Ebf1	6.74E-24	1.69165496	0.742	0.317	3.74E-19	Tnc_Mmp13_hiX
Limch1	7.79E-24	2.18513184	0.468	0.116	4.32E-19	Tnc_Mmp13_hiX
Gas6	1.15E-23	2.52005244	0.556	0.177	6.36E-19	Tnc_Mmp13_hiX
Srpx2	1.38E-23	1.65980475	0.548	0.168	7.67E-19	Tnc_Mmp13_hiX
Shox2	1.55E-23	1.59020656	0.597	0.215	8.56E-19	Tnc_Mmp13_hiX
Tmem176b	1.83E-23	1.52291577	0.815	0.577	1.01E-18	Tnc_Mmp13_hiX
Sema7a	2.14E-23	1.65312195	0.556	0.215	1.18E-18	Tnc_Mmp13_hiX
Palld	1.32E-22	1.52267849	0.476	0.17	7.34E-18	Tnc_Mmp13_hiX
Angpt4	1.63E-22	1.77297754	0.363	0.028	9.06E-18	Tnc_Mmp13_hiX
Col5a3	2.84E-22	1.75091617	0.331	0.021	1.57E-17	Tnc_Mmp13_hiX
Itgb5	7.79E-22	1.56041923	0.427	0.092	4.32E-17	Tnc_Mmp13_hiX
Gsn	8.74E-22	1.75366527	0.831	0.508	4.84E-17	Tnc_Mmp13_hiX
Alpl	1.64E-20	2.12745849	0.677	0.317	9.07E-16	Tnc_Mmp13_hiX
Vldlr	4.44E-20	1.9746534	0.435	0.175	2.46E-15	Tnc_Mmp13_hiX
Gpx3	4.55E-20	1.53574884	0.831	0.374	2.52E-15	Tnc_Mmp13_hiX
Ncam1	6.61E-20	1.66079517	0.565	0.284	3.66E-15	Tnc_Mmp13_hiX
Tnfrsf19	9.75E-20	1.55787396	0.387	0.109	5.40E-15	Tnc_Mmp13_hiX
Ccn5	1.64E-19	1.68055835	0.258	0.009	9.10E-15	Tnc_Mmp13_hiX
Pth1r	3.41E-18	1.53110125	0.774	0.35	1.89E-13	Tnc_Mmp13_hiX
Mir6236	7.45E-18	1.71157216	0.935	0.844	4.13E-13	Tnc_Mmp13_hiX
Wif1	2.52E-17	2.19119816	0.532	0.227	1.40E-12	Tnc_Mmp13_hiX
Cd109	1.01E-40	3.01657318	0.962	0.144	5.61E-36	Osteocytes
Phex	5.17E-39	3.90711405	1	0.19	2.87E-34	Osteocytes
Ackr3	1.17E-30	2.85849168	0.923	0.069	6.51E-26	Osteocytes
Pdgfa	1.07E-26	2.50221757	1	0.276	5.96E-22	Osteocytes
Irx5	2.20E-26	1.78314995	0.962	0.069	1.22E-21	Osteocytes
Pdpn	1.31E-24	2.28938698	0.885	0.067	7.27E-20	Osteocytes
Adamts14	1.38E-24	1.70433576	0.846	0.058	7.65E-20	Osteocytes
Dkk1	2.11E-24	2.54009464	0.923	0.067	1.17E-19	Osteocytes
Ramp1	3.70E-24	2.59364263	0.962	0.305	2.05E-19	Osteocytes
Ptprz1	6.19E-24	2.72814517	1	0.194	3.43E-19	Osteocytes
Car12	7.64E-23	2.61587306	0.731	0.035	4.23E-18	Osteocytes
Col24a1	2.73E-22	2.08837185	0.923	0.125	1.51E-17	Osteocytes
Bmp4	2.17E-21	1.80866938	0.885	0.077	1.20E-16	Osteocytes
Gm41724	9.69E-20	1.94281138	0.692	0.052	5.37E-15	Osteocytes
Wasl	1.01E-19	2.17987121	0.962	0.273	5.59E-15	Osteocytes
Ccdc194	1.45E-19	1.5658339	0.885	0.104	8.03E-15	Osteocytes
Bmp2	1.51E-19	2.05433911	0.846	0.113	8.36E-15	Osteocytes
Bambi	2.68E-19	2.55474561	0.923	0.386	1.48E-14	Osteocytes

Dmp1	4.84E-19	3.65905173	0.923	0.211	2.68E-14	Osteocytes
Spns2	1.41E-18	1.83744552	0.923	0.273	7.79E-14	Osteocytes
Ptgis	1.02E-17	2.05615892	1	0.307	5.67E-13	Osteocytes
Ccn4	1.11E-17	2.03981406	1	0.307	6.14E-13	Osteocytes
Myo1b	1.32E-17	1.71154411	0.962	0.338	7.29E-13	Osteocytes
Plpp1	1.94E-16	1.80693966	1	0.246	1.07E-11	Osteocytes
Cspg4	4.65E-16	1.5793323	0.923	0.171	2.58E-11	Osteocytes
Smpd3	2.49E-80	3.45806743	0.973	0.108	1.38E-75	Osteoblasts
Col11a2	2.12E-74	3.26461867	1	0.165	1.17E-69	Osteoblasts
Col1a1	5.04E-68	3.15036672	1	0.547	2.79E-63	Osteoblasts
Col11a1	2.06E-61	2.76005677	0.991	0.243	1.14E-56	Osteoblasts
Col5a2	2.23E-60	2.38841722	1	0.378	1.24E-55	Osteoblasts
Col1a2	6.04E-60	2.81528597	1	0.499	3.35E-55	Osteoblasts
Cpz	7.01E-59	2.09200578	0.855	0.078	3.88E-54	Osteoblasts
Col22a1	4.13E-58	1.83655941	0.973	0.176	2.29E-53	Osteoblasts
Col5a1	4.15E-58	2.13047401	0.991	0.281	2.30E-53	Osteoblasts
Cthrc1	1.40E-57	2.41654235	0.855	0.11	7.77E-53	Osteoblasts
Rrbp1	1.82E-57	1.92418914	0.991	0.627	1.01E-52	Osteoblasts
Serpinf1	1.23E-56	2.67505724	0.973	0.307	6.81E-52	Osteoblasts
Sparc	1.89E-56	2.35162287	1	0.65	1.05E-51	Osteoblasts
Fkbp11	4.01E-55	1.92949118	0.873	0.128	2.22E-50	Osteoblasts
Cgref1	3.49E-54	1.96338088	0.827	0.101	1.93E-49	Osteoblasts
Ifitm5	1.04E-52	2.2852029	0.873	0.126	5.79E-48	Osteoblasts
Bglap2	4.46E-50	3.33120685	0.955	0.286	2.47E-45	Osteoblasts
Bglap	7.46E-50	3.36374944	0.973	0.384	4.14E-45	Osteoblasts
Dcn	1.15E-49	1.57418471	0.991	0.277	6.37E-45	Osteoblasts
Creb3l1	2.24E-49	1.84217049	0.891	0.185	1.24E-44	Osteoblasts
Snhg18	6.37E-49	1.69881471	0.945	0.211	3.53E-44	Osteoblasts
Tmem119	2.71E-48	1.70493619	0.836	0.119	1.50E-43	Osteoblasts
Serpinh1	9.59E-48	1.85471201	1	0.558	5.32E-43	Osteoblasts
Lox	4.60E-47	1.65132074	0.927	0.208	2.55E-42	Osteoblasts
Car3	2.31E-45	1.80333235	0.909	0.19	1.28E-40	Osteoblasts
Rcn3	2.16E-43	1.64525629	0.982	0.437	1.19E-38	Osteoblasts
Gpx3	3.97E-43	1.56689112	1	0.346	2.20E-38	Osteoblasts
Tent5a	1.21E-42	1.73445597	0.936	0.323	6.70E-38	Osteoblasts
Kdelr2	1.71E-42	1.67049892	0.927	0.476	9.47E-38	Osteoblasts
Cdo1	5.64E-42	1.6974857	0.755	0.098	3.12E-37	Osteoblasts
Ibsp	5.82E-42	2.3490186	0.982	0.316	3.22E-37	Osteoblasts
Nupr1	2.56E-38	1.93815086	0.945	0.334	1.42E-33	Osteoblasts
Pcolce	3.90E-38	1.67779027	0.936	0.297	2.16E-33	Osteoblasts
Timp1	1.94E-27	1.85464172	0.791	0.238	1.07E-22	Osteoblasts
Ccn1	1.26E-20	2.08071419	0.718	0.256	6.98E-16	Osteoblasts
Cebpb	4.38E-103	4.25581685	1	0.534	2.43E-98	Hemopoetic_cells_II
Fth1	1.03E-100	4.0957679	1	0.908	5.73E-96	Hemopoetic_cells_II
Srgn	1.65E-91	4.51508538	0.987	0.41	9.13E-87	Hemopoetic_cells_II
Clec4d	6.15E-87	4.68182441	0.975	0.024	3.41E-82	Hemopoetic_cells_II

S100a11	1.32E-86	3.21154805	0.987	0.69	7.29E-82	Hemopoetic_cells_II
Il1r2	7.37E-85	5.00927235	0.975	0.043	4.09E-80	Hemopoetic_cells_II
Cxcl2	1.41E-83	6.57677754	0.987	0.077	7.81E-79	Hemopoetic_cells_II
Litaf	1.74E-79	3.06617092	0.924	0.498	9.65E-75	Hemopoetic_cells_II
Acod1	2.77E-79	5.43891476	0.924	0.019	1.54E-74	Hemopoetic_cells_II
Msrb1	4.18E-77	3.72521975	0.962	0.301	2.32E-72	Hemopoetic_cells_II
Slpi	4.93E-77	4.89034816	0.924	0.137	2.73E-72	Hemopoetic_cells_II
Hdc	9.25E-77	4.09871652	0.937	0.068	5.13E-72	Hemopoetic_cells_II
Il1b	5.55E-74	6.05420934	0.899	0.021	3.07E-69	Hemopoetic_cells_II
Grina	4.07E-73	3.27490485	0.911	0.404	2.26E-68	Hemopoetic_cells_II
Mcl1	8.99E-73	2.99848614	0.975	0.615	4.98E-68	Hemopoetic_cells_II
Trem1	3.36E-72	3.58033135	0.962	0.056	1.86E-67	Hemopoetic_cells_II
Ccr1	3.29E-71	3.79297571	0.899	0.049	1.82E-66	Hemopoetic_cells_II
Lmnb1	3.54E-66	3.56938076	0.949	0.154	1.96E-61	Hemopoetic_cells_II
Hcar2	5.57E-65	4.0909905	0.886	0.053	3.08E-60	Hemopoetic_cells_II
Clec4e	7.68E-65	3.98966993	0.911	0.049	4.25E-60	Hemopoetic_cells_II
Tyrobp	1.24E-64	3.73668	0.975	0.137	6.85E-60	Hemopoetic_cells_II
Nlrp3	1.17E-63	4.37416336	0.797	0.011	6.48E-59	Hemopoetic_cells_II
Clec7a	8.52E-61	4.17676701	0.823	0.034	4.72E-56	Hemopoetic_cells_II
Thbs1	8.84E-61	3.80349703	0.937	0.335	4.90E-56	Hemopoetic_cells_II
Csf3r	1.05E-60	3.48140018	0.835	0.043	5.83E-56	Hemopoetic_cells_II
Tpd52	5.18E-59	3.26118976	0.835	0.145	2.87E-54	Hemopoetic_cells_II
Slc16a3	2.36E-58	3.45348775	0.823	0.06	1.31E-53	Hemopoetic_cells_II
S100a8	2.87E-58	2.98499703	0.987	0.141	1.59E-53	Hemopoetic_cells_II
Txn1	1.69E-57	2.23800497	0.975	0.684	9.39E-53	Hemopoetic_cells_II
Adam8	9.01E-57	3.41605983	0.835	0.064	4.99E-52	Hemopoetic_cells_II
Btg1	1.38E-55	2.68339957	0.987	0.797	7.67E-51	Hemopoetic_cells_II
Ubc	4.19E-55	2.55052477	0.975	0.816	2.32E-50	Hemopoetic_cells_II
Plek	5.76E-55	4.03633402	0.823	0.098	3.19E-50	Hemopoetic_cells_II
G0s2	1.65E-54	4.20815846	0.873	0.103	9.16E-50	Hemopoetic_cells_II
Cd44	2.60E-54	3.15030631	0.937	0.331	1.44E-49	Hemopoetic_cells_II
S100a9	2.61E-54	3.18510418	0.962	0.171	1.45E-49	Hemopoetic_cells_II
Cd14	2.92E-53	4.24569867	0.797	0.124	1.62E-48	Hemopoetic_cells_II
Ndel1	5.46E-53	2.42889925	0.747	0.395	3.02E-48	Hemopoetic_cells_II
Cstb	8.42E-53	3.15875883	0.785	0.412	4.66E-48	Hemopoetic_cells_II
Rab7	2.12E-52	2.46519668	0.911	0.56	1.17E-47	Hemopoetic_cells_II
Taldo1	3.36E-52	2.25997083	0.899	0.59	1.86E-47	Hemopoetic_cells_II
Ftl1	9.97E-52	2.37950609	0.962	0.806	5.52E-47	Hemopoetic_cells_II
Ccl6	1.59E-51	3.77731872	0.722	0.026	8.83E-47	Hemopoetic_cells_II
Ptafr	1.90E-51	3.31094117	0.734	0.021	1.05E-46	Hemopoetic_cells_II
Mxd1	6.25E-50	2.86738475	0.747	0.109	3.46E-45	Hemopoetic_cells_II
Slc15a3	1.67E-49	2.87500552	0.709	0.034	9.26E-45	Hemopoetic_cells_II
Ccrl2	3.95E-49	4.10427128	0.747	0.085	2.19E-44	Hemopoetic_cells_II
Samsn1	7.34E-49	2.77822927	0.873	0.113	4.07E-44	Hemopoetic_cells_II
Ets2	8.49E-49	2.77291663	0.949	0.327	4.70E-44	Hemopoetic_cells_II
Cyp4f18	1.18E-48	3.05856893	0.709	0.021	6.54E-44	Hemopoetic_cells_II

Nfkbia	1.54E-48	3.05534098	0.911	0.566	8.51E-44	Hemopoetic_cells_II
Arg2	4.58E-48	2.97828305	0.671	0.019	2.54E-43	Hemopoetic_cells_II
Rnf149	6.19E-48	2.90353235	0.709	0.088	3.43E-43	Hemopoetic_cells_II
Ifrd1	7.04E-48	3.20905005	0.81	0.333	3.90E-43	Hemopoetic_cells_II
S100a6	9.10E-48	3.12664654	0.949	0.532	5.04E-43	Hemopoetic_cells_II
Pim1	1.06E-47	3.36075712	0.873	0.252	5.86E-43	Hemopoetic_cells_II
Card19	1.36E-47	2.82583804	0.734	0.254	7.55E-43	Hemopoetic_cells_II
Ninj1	5.98E-47	3.08967248	0.759	0.269	3.31E-42	Hemopoetic_cells_II
Retnlg	6.36E-47	5.09094351	0.797	0.073	3.52E-42	Hemopoetic_cells_II
Alox5ap	6.75E-47	2.75229496	0.886	0.113	3.74E-42	Hemopoetic_cells_II
Gsr	2.78E-46	2.40744576	0.823	0.406	1.54E-41	Hemopoetic_cells_II
Plaur	1.30E-45	2.89956032	0.861	0.214	7.19E-41	Hemopoetic_cells_II
Ptprc	5.40E-45	2.70685244	0.873	0.132	2.99E-40	Hemopoetic_cells_II
Cd53	6.87E-45	2.81989038	0.848	0.139	3.81E-40	Hemopoetic_cells_II
Lsp1	1.27E-44	2.61434396	0.835	0.184	7.07E-40	Hemopoetic_cells_II
Fcer1g	4.75E-43	2.57200446	0.937	0.321	2.63E-38	Hemopoetic_cells_II
Stx11	6.41E-43	2.82919052	0.671	0.062	3.55E-38	Hemopoetic_cells_II
Prdx5	1.36E-42	2.05850369	0.886	0.618	7.53E-38	Hemopoetic_cells_II
Pnrc1	2.23E-42	2.1147655	0.886	0.579	1.23E-37	Hemopoetic_cells_II
Emilin2	6.05E-42	2.45790397	0.633	0.03	3.35E-37	Hemopoetic_cells_II
H3f3b	8.85E-42	1.86457824	0.975	0.897	4.91E-37	Hemopoetic_cells_II
Mrpl33	1.73E-41	1.65769172	0.684	0.532	9.59E-37	Hemopoetic_cells_II
Vps37b	2.02E-41	3.30159967	0.734	0.109	1.12E-36	Hemopoetic_cells_II
Il1rn	3.92E-41	3.80682143	0.696	0.045	2.17E-36	Hemopoetic_cells_II
Mmp9	4.22E-41	1.62365172	0.848	0.103	2.34E-36	Hemopoetic_cells_II
Cxcr4	4.24E-41	3.22932401	0.772	0.124	2.35E-36	Hemopoetic_cells_II
Rab8b	6.62E-41	2.42650651	0.734	0.276	3.67E-36	Hemopoetic_cells_II
Cdk2ap2	1.06E-40	2.43515249	0.785	0.344	5.88E-36	Hemopoetic_cells_II
Eif1	1.14E-40	1.56935076	0.975	0.88	6.32E-36	Hemopoetic_cells_II
Ccl3	1.82E-40	4.61367284	0.722	0.062	1.01E-35	Hemopoetic_cells_II
C5ar1	4.21E-40	2.82640663	0.734	0.081	2.33E-35	Hemopoetic_cells_II
Cd52	4.42E-40	2.67454159	0.797	0.12	2.45E-35	Hemopoetic_cells_II
Arpc3	6.79E-40	2.01806398	0.899	0.596	3.76E-35	Hemopoetic_cells_II
Selenok	1.14E-39	1.63683017	0.861	0.782	6.32E-35	Hemopoetic_cells_II
Antxr2	1.71E-39	2.06432561	0.671	0.382	9.50E-35	Hemopoetic_cells_II
Pik3ap1	5.63E-39	2.7908406	0.62	0.053	3.12E-34	Hemopoetic_cells_II
Lst1	1.72E-38	2.97553269	0.759	0.137	9.56E-34	Hemopoetic_cells_II
Ncf2	2.01E-38	2.5113756	0.709	0.098	1.11E-33	Hemopoetic_cells_II
Efhd2	2.27E-38	2.49816777	0.81	0.233	1.26E-33	Hemopoetic_cells_II
Tlr2	2.83E-38	2.83856522	0.646	0.075	1.57E-33	Hemopoetic_cells_II
Rabgef1	8.69E-38	2.22176865	0.646	0.105	4.82E-33	Hemopoetic_cells_II
Sorl1	1.27E-37	2.48428643	0.709	0.083	7.04E-33	Hemopoetic_cells_II
Tgoln1	3.69E-37	1.98490575	0.696	0.498	2.04E-32	Hemopoetic_cells_II
Pglyrp1	5.21E-37	2.38600529	0.772	0.083	2.89E-32	Hemopoetic_cells_II
Map1lc3b	7.28E-37	1.68244232	0.861	0.682	4.03E-32	Hemopoetic_cells_II
Rac2	1.73E-36	2.19978992	0.873	0.162	9.58E-32	Hemopoetic_cells_II

Coro1a	8.91E-36	2.28345546	0.861	0.152	4.94E-31	Hemopoetic_cells_II
Nfkbiz	8.98E-36	2.64353797	0.797	0.278	4.98E-31	Hemopoetic_cells_II
Dgat1	2.57E-35	2.83439382	0.684	0.192	1.42E-30	Hemopoetic_cells_II
Cd300lf	3.33E-35	2.30651237	0.62	0.045	1.84E-30	Hemopoetic_cells_II
Bcl2l11	7.02E-35	2.37876503	0.57	0.145	3.89E-30	Hemopoetic_cells_II
Tnfrsf1b	7.71E-35	2.50850001	0.608	0.103	4.27E-30	Hemopoetic_cells_II
Lcp1	1.00E-34	2.07293235	0.873	0.259	5.56E-30	Hemopoetic_cells_II
Vasp	1.28E-34	2.10167215	0.785	0.436	7.11E-30	Hemopoetic_cells_II
Dusp1	2.07E-34	2.39214103	0.962	0.464	1.15E-29	Hemopoetic_cells_II
Cd9	2.60E-34	1.82224706	0.937	0.765	1.44E-29	Hemopoetic_cells_II
Trib1	2.66E-34	2.34063051	0.797	0.214	1.47E-29	Hemopoetic_cells_II
Dedd2	2.68E-34	2.61635319	0.582	0.092	1.49E-29	Hemopoetic_cells_II
Mcemp1	4.41E-34	2.26533904	0.696	0.066	2.44E-29	Hemopoetic_cells_II
Smim3	4.46E-34	2.25694029	0.633	0.118	2.47E-29	Hemopoetic_cells_II
Csf2ra	6.27E-34	2.00899078	0.671	0.126	3.48E-29	Hemopoetic_cells_II
Mirt2	9.49E-34	2.09360339	0.506	0.011	5.26E-29	Hemopoetic_cells_II
Il1f9	1.77E-33	2.91209434	0.506	0.009	9.80E-29	Hemopoetic_cells_II
Cd33	4.57E-33	2.18471687	0.595	0.045	2.53E-28	Hemopoetic_cells_II
Gadd45b	5.20E-33	2.55597766	0.823	0.432	2.88E-28	Hemopoetic_cells_II
Prr13	9.46E-33	2.08094161	0.696	0.323	5.24E-28	Hemopoetic_cells_II
Itgb2	1.03E-32	2.19176562	0.709	0.105	5.72E-28	Hemopoetic_cells_II
Ehd1	1.64E-32	2.1402682	0.684	0.34	9.09E-28	Hemopoetic_cells_II
Dazap2	2.12E-32	1.84073326	0.823	0.566	1.17E-27	Hemopoetic_cells_II
Smox	2.30E-32	2.02326373	0.595	0.278	1.27E-27	Hemopoetic_cells_II
Plk3	4.97E-32	2.3632877	0.671	0.137	2.76E-27	Hemopoetic_cells_II
Slfn2	8.09E-32	2.61179837	0.696	0.13	4.48E-27	Hemopoetic_cells_II
Kdm6b	9.52E-32	2.35283297	0.696	0.348	5.27E-27	Hemopoetic_cells_II
Asprv1	1.26E-31	3.56906349	0.481	0.015	6.96E-27	Hemopoetic_cells_II
Rab11fip1	1.40E-31	2.17951376	0.494	0.021	7.76E-27	Hemopoetic_cells_II
Selenon	1.49E-31	2.01693583	0.671	0.301	8.26E-27	Hemopoetic_cells_II
Ier5	2.14E-31	2.23682246	0.81	0.459	1.18E-26	Hemopoetic_cells_II
Gmfg	2.16E-31	2.23748588	0.734	0.192	1.20E-26	Hemopoetic_cells_II
Ifitm2	2.73E-31	1.88379074	0.722	0.6	1.52E-26	Hemopoetic_cells_II
Tax1bp1	4.32E-31	1.65002968	0.671	0.519	2.39E-26	Hemopoetic_cells_II
Fem1c	9.40E-31	1.89935779	0.608	0.177	5.21E-26	Hemopoetic_cells_II
Syk	1.24E-30	2.34172944	0.608	0.096	6.89E-26	Hemopoetic_cells_II
Tmsb4x	1.45E-30	1.76503229	1	0.932	8.01E-26	Hemopoetic_cells_II
Snx20	1.73E-30	2.1348357	0.532	0.056	9.59E-26	Hemopoetic_cells_II
Laptm5	2.05E-30	2.22690042	0.81	0.169	1.13E-25	Hemopoetic_cells_II
Cd24a	2.08E-30	2.18026888	0.797	0.192	1.15E-25	Hemopoetic_cells_II
Basp1	2.10E-30	3.37634388	0.557	0.132	1.17E-25	Hemopoetic_cells_II
Map2k3	2.97E-30	1.73317272	0.646	0.37	1.65E-25	Hemopoetic_cells_II
Cotl1	4.19E-30	1.99158106	0.797	0.35	2.32E-25	Hemopoetic_cells_II
Themis2	7.96E-30	1.81517488	0.532	0.038	4.41E-25	Hemopoetic_cells_II
Cd300ld	9.88E-30	2.03781577	0.456	0.013	5.47E-25	Hemopoetic_cells_II
Sh2d3c	1.76E-29	1.95815105	0.557	0.143	9.75E-25	Hemopoetic_cells_II

Fmnl1	3.36E-29	1.93066935	0.608	0.098	1.86E-24	Hemopoetic_cells_II
Anxa11	5.95E-29	1.64241616	0.608	0.331	3.30E-24	Hemopoetic_cells_II
Mapkapk2	7.58E-29	1.82789898	0.633	0.4	4.20E-24	Hemopoetic_cells_II
Wfdc21	9.08E-29	2.39321629	0.646	0.075	5.03E-24	Hemopoetic_cells_II
Atp6v1e1	1.09E-28	1.75687923	0.646	0.502	6.04E-24	Hemopoetic_cells_II
Pla2g7	1.44E-28	2.34216125	0.519	0.032	7.98E-24	Hemopoetic_cells_II
Slc9a3r1	1.46E-28	2.10859563	0.557	0.115	8.07E-24	Hemopoetic_cells_II
Lasp1	1.57E-28	1.84014311	0.709	0.472	8.70E-24	Hemopoetic_cells_II
H2-Q10	1.79E-28	2.1919859	0.456	0.024	9.90E-24	Hemopoetic_cells_II
Osgin1	2.30E-28	2.05399177	0.532	0.041	1.28E-23	Hemopoetic_cells_II
Iqgap1	2.70E-28	1.5677719	0.785	0.558	1.50E-23	Hemopoetic_cells_II
Tgfbi	3.52E-28	1.98445989	0.633	0.103	1.95E-23	Hemopoetic_cells_II
Trem3	3.85E-28	1.91527766	0.608	0.062	2.13E-23	Hemopoetic_cells_II
Mmp8	1.07E-27	2.74643465	0.557	0.041	5.92E-23	Hemopoetic_cells_II
Tnfaip2	1.15E-27	2.30548319	0.608	0.321	6.40E-23	Hemopoetic_cells_II
Jdp2	1.36E-27	1.93562962	0.633	0.291	7.53E-23	Hemopoetic_cells_II
Osm	3.39E-27	1.99323969	0.633	0.073	1.88E-22	Hemopoetic_cells_II
Cox17	5.53E-27	1.72614458	0.557	0.318	3.07E-22	Hemopoetic_cells_II
Gm20406	8.98E-27	2.29426442	0.506	0.038	4.98E-22	Hemopoetic_cells_II
Bcl2a1b	1.14E-26	2.65227445	0.468	0.032	6.29E-22	Hemopoetic_cells_II
Pilra	1.25E-26	1.95637352	0.582	0.079	6.92E-22	Hemopoetic_cells_II
Cyba	1.27E-26	1.52245646	0.886	0.682	7.03E-22	Hemopoetic_cells_II
Gla	1.93E-26	1.89146857	0.481	0.06	1.07E-21	Hemopoetic_cells_II
Adipor1	2.42E-26	1.68630211	0.658	0.442	1.34E-21	Hemopoetic_cells_II
Kpna4	2.76E-26	1.74323944	0.62	0.276	1.53E-21	Hemopoetic_cells_II
Nr4a3	2.87E-26	2.23969456	0.481	0.051	1.59E-21	Hemopoetic_cells_II
Tnfaip3	3.18E-26	2.57859393	0.595	0.115	1.76E-21	Hemopoetic_cells_II
Resf1	3.23E-26	1.8557602	0.658	0.395	1.79E-21	Hemopoetic_cells_II
Slc2a3	5.28E-26	2.24983547	0.544	0.066	2.92E-21	Hemopoetic_cells_II
Cstdc4	8.71E-26	3.69951749	0.367	0.002	4.83E-21	Hemopoetic_cells_II
Ankrd33b	1.10E-25	1.96993765	0.456	0.047	6.10E-21	Hemopoetic_cells_II
Irak2	1.16E-25	2.09937153	0.456	0.051	6.44E-21	Hemopoetic_cells_II
Ifitm1	1.32E-25	2.91209163	0.519	0.156	7.30E-21	Hemopoetic_cells_II
Hp	1.49E-25	1.79187791	0.734	0.145	8.24E-21	Hemopoetic_cells_II
Nfe2l2	1.84E-25	1.92751889	0.633	0.37	1.02E-20	Hemopoetic_cells_II
Hilpda	3.88E-25	2.12627366	0.392	0.229	2.15E-20	Hemopoetic_cells_II
Tgm2	3.89E-25	2.2654501	0.658	0.278	2.16E-20	Hemopoetic_cells_II
Tgif1	4.75E-25	2.07933078	0.494	0.16	2.63E-20	Hemopoetic_cells_II
Rab20	6.84E-25	2.04618052	0.519	0.073	3.79E-20	Hemopoetic_cells_II
Gfod1	7.14E-25	1.88409781	0.443	0.1	3.96E-20	Hemopoetic_cells_II
Fxyd5	1.07E-24	1.83707286	0.646	0.288	5.91E-20	Hemopoetic_cells_II
Glrx	1.50E-24	1.88773288	0.62	0.16	8.33E-20	Hemopoetic_cells_II
Marcksl1	2.16E-24	2.58423865	0.722	0.387	1.20E-19	Hemopoetic_cells_II
Stfa2l1	2.30E-24	3.03924933	0.38	0.009	1.28E-19	Hemopoetic_cells_II
Zfp36	3.64E-24	2.00734034	0.861	0.483	2.02E-19	Hemopoetic_cells_II
Spi1	4.82E-24	1.92792803	0.633	0.109	2.67E-19	Hemopoetic_cells_II

Hcst	6.62E-24	1.78883341	0.608	0.109	3.67E-19	Hemopoetic_cells_II
Lgals3	8.70E-24	1.92773671	0.684	0.143	4.82E-19	Hemopoetic_cells_II
2310001H17I	3.32E-23	1.72091541	0.418	0.032	1.84E-18	Hemopoetic_cells_II
Vegfa	4.36E-23	1.84723315	0.506	0.327	2.42E-18	Hemopoetic_cells_II
Pde4b	5.03E-23	2.08559188	0.582	0.12	2.79E-18	Hemopoetic_cells_II
Retreg1	5.51E-23	1.95265837	0.519	0.109	3.05E-18	Hemopoetic_cells_II
Sell	7.92E-23	2.16411918	0.506	0.064	4.39E-18	Hemopoetic_cells_II
Rhog	2.32E-22	1.50578137	0.519	0.259	1.29E-17	Hemopoetic_cells_II
Lrrc25	2.76E-22	1.61027972	0.405	0.053	1.53E-17	Hemopoetic_cells_II
Hcls1	2.99E-22	1.59819557	0.633	0.169	1.66E-17	Hemopoetic_cells_II
Dusp5	3.47E-22	2.33544776	0.544	0.128	1.92E-17	Hemopoetic_cells_II
Sephs2	4.26E-22	1.61937758	0.456	0.184	2.36E-17	Hemopoetic_cells_II
Chd7	1.21E-21	1.7618741	0.456	0.165	6.71E-17	Hemopoetic_cells_II
Inpp5d	2.13E-21	1.70577025	0.532	0.147	1.18E-16	Hemopoetic_cells_II
Ifi27l2a	3.44E-21	2.65498135	0.544	0.083	1.91E-16	Hemopoetic_cells_II
Sat1	4.02E-21	1.69207675	0.835	0.434	2.23E-16	Hemopoetic_cells_II
Plbd1	4.22E-21	1.68942179	0.608	0.105	2.34E-16	Hemopoetic_cells_II
Rdh12	5.71E-21	1.60950863	0.354	0.019	3.16E-16	Hemopoetic_cells_II
Rhov	6.97E-21	1.53165575	0.291	0.002	3.86E-16	Hemopoetic_cells_II
1700017B05I	1.14E-20	1.66813955	0.405	0.098	6.33E-16	Hemopoetic_cells_II
Rasa2	2.10E-20	1.83245288	0.494	0.205	1.16E-15	Hemopoetic_cells_II
Lyst	2.41E-20	1.60578956	0.506	0.218	1.33E-15	Hemopoetic_cells_II
Suco	2.41E-20	1.69050604	0.519	0.207	1.34E-15	Hemopoetic_cells_II
Marchf7	2.69E-20	1.5448417	0.532	0.316	1.49E-15	Hemopoetic_cells_II
Hdac4	4.27E-20	1.63328215	0.456	0.169	2.37E-15	Hemopoetic_cells_II
Ncf4	7.24E-20	1.59936752	0.519	0.094	4.01E-15	Hemopoetic_cells_II
Lfng	8.23E-20	1.81127611	0.43	0.16	4.56E-15	Hemopoetic_cells_II
Zswim4	1.00E-19	1.59236872	0.342	0.071	5.56E-15	Hemopoetic_cells_II
Selplg	1.06E-19	1.66284124	0.418	0.06	5.88E-15	Hemopoetic_cells_II
Rilpl2	1.60E-19	1.87346887	0.367	0.184	8.87E-15	Hemopoetic_cells_II
Pygl	2.39E-19	1.7120359	0.494	0.077	1.33E-14	Hemopoetic_cells_II
Il17ra	2.80E-19	1.5195927	0.405	0.083	1.55E-14	Hemopoetic_cells_II
Fosl2	2.92E-19	1.68594567	0.658	0.316	1.62E-14	Hemopoetic_cells_II
Zc3h12a	7.35E-19	2.00716138	0.456	0.098	4.07E-14	Hemopoetic_cells_II
Snx18	9.35E-19	1.50407792	0.582	0.231	5.18E-14	Hemopoetic_cells_II
Entpd1	1.83E-18	1.77907494	0.443	0.137	1.02E-13	Hemopoetic_cells_II
Ptk2b	2.36E-18	1.77748964	0.367	0.064	1.31E-13	Hemopoetic_cells_II
Tnf	2.90E-18	2.39063114	0.456	0.068	1.61E-13	Hemopoetic_cells_II
Fgd3	3.11E-18	1.60433272	0.304	0.034	1.72E-13	Hemopoetic_cells_II
Zfp263	4.29E-18	1.54774453	0.316	0.137	2.38E-13	Hemopoetic_cells_II
Prdx6	5.71E-18	1.56290377	0.658	0.457	3.16E-13	Hemopoetic_cells_II
Ezr	2.38E-17	1.54290817	0.481	0.188	1.32E-12	Hemopoetic_cells_II
Sqstm1	2.52E-17	1.50728253	0.392	0.436	1.40E-12	Hemopoetic_cells_II
Cytip	3.30E-17	1.64241095	0.43	0.071	1.83E-12	Hemopoetic_cells_II
Tiparp	3.77E-17	1.72534791	0.532	0.261	2.09E-12	Hemopoetic_cells_II
Slc7a11	4.09E-17	1.53973764	0.304	0.013	2.27E-12	Hemopoetic_cells_II

Mreg	4.69E-17	2.07130288	0.278	0.013	2.60E-12	Hemopoetic_cells_II
Ccl4	4.95E-17	3.16699059	0.443	0.058	2.74E-12	Hemopoetic_cells_II
AB124611	5.94E-17	1.54508115	0.456	0.092	3.29E-12	Hemopoetic_cells_II
Gadd45a	6.07E-17	1.59480567	0.633	0.188	3.36E-12	Hemopoetic_cells_II
Birc3	8.21E-17	1.66797353	0.38	0.1	4.55E-12	Hemopoetic_cells_II
Neurl3	1.03E-16	1.81654065	0.367	0.049	5.73E-12	Hemopoetic_cells_II
Nfkbid	1.05E-16	1.67294072	0.405	0.071	5.80E-12	Hemopoetic_cells_II
Gda	4.11E-16	1.53339551	0.519	0.192	2.28E-11	Hemopoetic_cells_II
Ptgs2	5.80E-16	2.04151873	0.304	0.038	3.21E-11	Hemopoetic_cells_II
Rgs2	6.90E-16	1.68147567	0.62	0.361	3.83E-11	Hemopoetic_cells_II
Rel	9.36E-16	1.85159241	0.405	0.173	5.19E-11	Hemopoetic_cells_II
Fpr1	1.28E-15	1.65397168	0.316	0.024	7.11E-11	Hemopoetic_cells_II
Bhlhe40	1.64E-15	1.57739637	0.532	0.288	9.10E-11	Hemopoetic_cells_II
H2az1	4.50E-30	1.766476	0.637	0.312	2.49E-25	Hemopoetic_cells_I
Cybb	8.28E-29	3.03963607	0.51	0.056	4.59E-24	Hemopoetic_cells_I
Chil3	5.49E-25	4.09821122	0.343	0.011	3.05E-20	Hemopoetic_cells_I
Gpx1	1.14E-22	1.62903716	0.873	0.622	6.30E-18	Hemopoetic_cells_I
Ltf	3.68E-22	3.51742142	0.284	0.013	2.04E-17	Hemopoetic_cells_I
Ptpn18	3.68E-22	1.58402554	0.618	0.146	2.04E-17	Hemopoetic_cells_I
Tkt	5.28E-22	1.72660827	0.676	0.407	2.92E-17	Hemopoetic_cells_I
Hmgb2	5.72E-22	2.07935635	0.716	0.344	3.17E-17	Hemopoetic_cells_I
Arhgdib	1.23E-21	1.94924761	0.716	0.24	6.81E-17	Hemopoetic_cells_I
Coro1a	2.13E-21	1.60303314	0.657	0.162	1.18E-16	Hemopoetic_cells_I
Cd74	9.94E-21	2.84919195	0.294	0.007	5.51E-16	Hemopoetic_cells_I
Camp	1.75E-20	6.21131503	0.363	0.058	9.69E-16	Hemopoetic_cells_I
Clec12a	2.24E-19	1.53144918	0.373	0.031	1.24E-14	Hemopoetic_cells_I
Mki67	8.50E-18	1.91602355	0.324	0.025	4.71E-13	Hemopoetic_cells_I
Ngp	1.19E-17	5.01470869	0.314	0.076	6.61E-13	Hemopoetic_cells_I
Ighm	1.53E-17	2.29795404	0.304	0.031	8.46E-13	Hemopoetic_cells_I
Birc5	1.77E-17	1.54245633	0.245	0.007	9.78E-13	Hemopoetic_cells_I
Plac8	2.70E-17	2.29557516	0.382	0.065	1.50E-12	Hemopoetic_cells_I
Top2a	2.86E-17	1.74393053	0.275	0.016	1.59E-12	Hemopoetic_cells_I
H2-Ab1	1.19E-16	3.16433898	0.314	0.036	6.58E-12	Hemopoetic_cells_I
Ctss	2.12E-16	2.04839286	0.294	0.02	1.18E-11	Hemopoetic_cells_I
Lyz2	7.76E-16	3.0399075	0.549	0.18	4.30E-11	Hemopoetic_cells_I
Stmn1	1.50E-15	1.89266974	0.333	0.09	8.32E-11	Hemopoetic_cells_I
Cdh5	1.29E-107	3.9470637	0.972	0.014	7.16E-103	Bone marrow endothelial cells
Plvap	6.27E-104	4.09682951	0.953	0.036	3.48E-99	Bone marrow endothelial cells
Ptprb	1.12E-101	3.65970704	0.953	0.007	6.21E-97	Bone marrow endothelial cells
Sox18	1.19E-100	3.5804856	0.943	0.014	6.59E-96	Bone marrow endothelial cells
Esam	1.16E-99	3.36955146	0.972	0.018	6.44E-95	Bone marrow endothelial cells
Pecam1	1.75E-99	3.35685611	0.972	0.016	9.71E-95	Bone marrow endothelial cells
Mmrn2	5.22E-97	2.91393495	0.934	0.007	2.89E-92	Bone marrow endothelial cells
Tie1	2.05E-94	2.67116947	0.934	0.011	1.14E-89	Bone marrow endothelial cells
Egfl7	4.76E-94	3.14008023	0.925	0.009	2.64E-89	Bone marrow endothelial cells
Kdr	3.60E-93	3.40613254	0.925	0.014	2.00E-88	Bone marrow endothelial cells

Eng	7.31E-90	3.34354008	0.962	0.136	4.05E-85	Bone marrow endothelial cells
Cldn5	2.71E-89	4.31608054	0.906	0.011	1.50E-84	Bone marrow endothelial cells
Rasip1	4.03E-89	2.38497293	0.953	0.027	2.23E-84	Bone marrow endothelial cells
Emcn	2.48E-88	2.97380724	0.887	0.007	1.38E-83	Bone marrow endothelial cells
Flt1	4.34E-88	2.98676068	0.887	0.007	2.40E-83	Bone marrow endothelial cells
Ecscr	1.12E-86	2.5277791	0.887	0.016	6.23E-82	Bone marrow endothelial cells
F11r	4.08E-86	2.37732416	0.877	0.02	2.26E-81	Bone marrow endothelial cells
Ly6a	6.22E-83	3.95806914	0.906	0.036	3.45E-78	Bone marrow endothelial cells
Gng11	6.72E-83	3.20956388	0.953	0.308	3.72E-78	Bone marrow endothelial cells
Ushbp1	2.74E-82	2.1931432	0.83	0.002	1.52E-77	Bone marrow endothelial cells
Cd93	2.13E-81	2.94644321	0.962	0.091	1.18E-76	Bone marrow endothelial cells
Podxl	2.32E-77	3.01504293	0.792	0.002	1.29E-72	Bone marrow endothelial cells
Tcim	7.58E-77	3.58009375	0.858	0.034	4.20E-72	Bone marrow endothelial cells
Ldb2	9.72E-75	2.21495619	0.821	0.016	5.39E-70	Bone marrow endothelial cells
Ets1	1.04E-74	2.69437825	0.943	0.168	5.78E-70	Bone marrow endothelial cells
Icam2	1.94E-74	2.62345053	0.849	0.032	1.08E-69	Bone marrow endothelial cells
Adgrl4	3.35E-72	2.07052863	0.792	0.009	1.86E-67	Bone marrow endothelial cells
Myct1	1.43E-71	2.20971349	0.811	0.016	7.90E-67	Bone marrow endothelial cells
Tek	4.16E-71	2.03129646	0.764	0.005	2.30E-66	Bone marrow endothelial cells
Npr1	2.55E-70	2.17447172	0.792	0.016	1.42E-65	Bone marrow endothelial cells
Robo4	7.18E-70	2.07412049	0.755	0.005	3.98E-65	Bone marrow endothelial cells
Cd81	1.15E-69	2.46724925	0.972	0.533	6.35E-65	Bone marrow endothelial cells
Epas1	1.03E-68	2.73021914	0.972	0.186	5.68E-64	Bone marrow endothelial cells
Sptbn1	2.03E-68	2.41885348	0.972	0.376	1.12E-63	Bone marrow endothelial cells
Ctla2a	2.43E-68	3.51066372	0.811	0.027	1.35E-63	Bone marrow endothelial cells
Clec14a	1.18E-67	2.68718781	0.811	0.029	6.53E-63	Bone marrow endothelial cells
Fam167b	1.29E-67	2.29907643	0.745	0.009	7.13E-63	Bone marrow endothelial cells
Fkbp1a	1.33E-67	2.20890014	0.934	0.449	7.38E-63	Bone marrow endothelial cells
Adgrf5	1.86E-66	2.36895824	0.915	0.102	1.03E-61	Bone marrow endothelial cells
Slc9a3r2	1.62E-64	3.05664499	0.821	0.104	8.98E-60	Bone marrow endothelial cells
Tm4sf1	3.12E-64	3.49514761	0.896	0.229	1.73E-59	Bone marrow endothelial cells
Col4a1	3.85E-64	2.93910665	0.906	0.093	2.13E-59	Bone marrow endothelial cells
Rapgef5	5.46E-64	1.87547772	0.717	0.007	3.03E-59	Bone marrow endothelial cells
Arhgef15	1.05E-63	1.77456703	0.717	0.007	5.83E-59	Bone marrow endothelial cells
Flt4	3.85E-63	2.39601201	0.698	0.007	2.13E-58	Bone marrow endothelial cells
Fgd5	5.59E-63	1.76118192	0.736	0.018	3.10E-58	Bone marrow endothelial cells
Cavin3	6.38E-63	2.37553309	0.934	0.265	3.53E-58	Bone marrow endothelial cells
Ece1	7.37E-63	2.22329273	0.906	0.193	4.08E-58	Bone marrow endothelial cells
Ly6e	9.52E-63	2.50354202	0.981	0.265	5.28E-58	Bone marrow endothelial cells
Ccdc85b	1.17E-62	2.22823805	0.868	0.24	6.49E-58	Bone marrow endothelial cells
Jup	1.53E-62	2.12003582	0.83	0.118	8.47E-58	Bone marrow endothelial cells
Mcam	2.65E-61	2.23240325	0.764	0.025	1.47E-56	Bone marrow endothelial cells
Elk3	1.18E-60	2.14903504	0.849	0.197	6.52E-56	Bone marrow endothelial cells
Col4a2	3.76E-60	2.9351353	0.858	0.084	2.09E-55	Bone marrow endothelial cells
Arhgap29	1.18E-59	1.85400134	0.783	0.039	6.55E-55	Bone marrow endothelial cells
Plpp3	2.72E-59	2.36937354	0.906	0.109	1.51E-54	Bone marrow endothelial cells

Cavin2	4.49E-59	2.33272253	0.679	0.009	2.49E-54	Bone marrow endothelial cells
Thbd	7.32E-59	2.49703341	0.745	0.034	4.06E-54	Bone marrow endothelial cells
Cd34	3.66E-57	2.77997977	0.708	0.023	2.03E-52	Bone marrow endothelial cells
She	4.31E-57	1.58066828	0.66	0.009	2.39E-52	Bone marrow endothelial cells
Plpp1	5.04E-57	2.21836968	0.906	0.132	2.79E-52	Bone marrow endothelial cells
Fabp4	7.98E-57	5.0919154	0.708	0.032	4.42E-52	Bone marrow endothelial cells
Plxnd1	8.37E-57	2.33526766	0.906	0.177	4.64E-52	Bone marrow endothelial cells
Slfn5	3.01E-56	2.14892774	0.868	0.093	1.67E-51	Bone marrow endothelial cells
Rasgrp3	1.24E-55	1.52466592	0.632	0.005	6.90E-51	Bone marrow endothelial cells
Crip2	5.43E-55	2.2193365	0.915	0.311	3.01E-50	Bone marrow endothelial cells
Btnl9	8.40E-55	2.17010206	0.613	0.002	4.65E-50	Bone marrow endothelial cells
Timp3	8.87E-55	2.52936426	0.915	0.195	4.91E-50	Bone marrow endothelial cells
Adgrg1	9.31E-55	1.53676376	0.66	0.016	5.16E-50	Bone marrow endothelial cells
Shank3	9.64E-55	1.50304355	0.67	0.014	5.34E-50	Bone marrow endothelial cells
Hspb1	1.40E-54	2.6597172	0.811	0.066	7.78E-50	Bone marrow endothelial cells
Rhoj	1.43E-54	1.77218535	0.783	0.059	7.95E-50	Bone marrow endothelial cells
Ehd2	1.79E-53	1.93127055	0.774	0.07	9.92E-49	Bone marrow endothelial cells
Dll4	2.74E-53	1.93249814	0.613	0.005	1.52E-48	Bone marrow endothelial cells
Tmem88	5.89E-53	1.66443157	0.66	0.02	3.26E-48	Bone marrow endothelial cells
Itga6	1.38E-52	1.73593642	0.679	0.025	7.67E-48	Bone marrow endothelial cells
Ltbp4	2.79E-52	2.9027576	0.717	0.043	1.55E-47	Bone marrow endothelial cells
Igfbp7	4.34E-52	2.70328338	0.915	0.17	2.41E-47	Bone marrow endothelial cells
Tcf4	1.06E-51	1.89771192	0.981	0.465	5.85E-47	Bone marrow endothelial cells
Cracr2b	1.72E-51	1.65541559	0.67	0.032	9.53E-47	Bone marrow endothelial cells
Prex2	4.45E-51	1.65918499	0.594	0.005	2.47E-46	Bone marrow endothelial cells
C130074G19I	6.88E-51	1.64858989	0.651	0.018	3.81E-46	Bone marrow endothelial cells
Pcdh17	1.14E-50	1.75803136	0.632	0.014	6.32E-46	Bone marrow endothelial cells
Fzd4	3.23E-50	1.60989725	0.736	0.054	1.79E-45	Bone marrow endothelial cells
Selenop	7.36E-50	2.954117	0.925	0.417	4.08E-45	Bone marrow endothelial cells
Cmtm8	8.25E-50	1.72965999	0.632	0.025	4.57E-45	Bone marrow endothelial cells
Sox17	9.43E-50	2.03903183	0.585	0.007	5.22E-45	Bone marrow endothelial cells
Calcrl	2.50E-49	1.70461827	0.679	0.039	1.38E-44	Bone marrow endothelial cells
Cd300lg	3.44E-48	2.11942045	0.66	0.029	1.91E-43	Bone marrow endothelial cells
Abcg2	5.82E-48	1.63041288	0.689	0.059	3.22E-43	Bone marrow endothelial cells
Stab1	1.84E-47	1.81243798	0.651	0.025	1.02E-42	Bone marrow endothelial cells
Hspg2	2.22E-47	1.91537068	0.934	0.472	1.23E-42	Bone marrow endothelial cells
Gimap4	7.06E-47	1.58255663	0.604	0.014	3.91E-42	Bone marrow endothelial cells
2900026A02I	2.43E-46	1.58493915	0.755	0.088	1.35E-41	Bone marrow endothelial cells
Cd38	4.46E-46	1.6170629	0.623	0.02	2.47E-41	Bone marrow endothelial cells
Serpinb6a	1.99E-45	1.92700528	0.896	0.32	1.10E-40	Bone marrow endothelial cells
Cyb5a	2.98E-45	1.73165146	0.896	0.277	1.65E-40	Bone marrow endothelial cells
Arap3	3.10E-45	1.63974896	0.708	0.07	1.72E-40	Bone marrow endothelial cells
Ifitm3	3.29E-45	1.93172165	0.981	0.66	1.82E-40	Bone marrow endothelial cells
Notch4	6.38E-45	1.59856067	0.623	0.043	3.54E-40	Bone marrow endothelial cells
Slc43a3	4.08E-44	1.57291727	0.67	0.05	2.26E-39	Bone marrow endothelial cells
Cav1	5.91E-44	1.85351358	0.585	0.018	3.28E-39	Bone marrow endothelial cells

Crim1	9.17E-44	1.72473586	0.783	0.175	5.08E-39	Bone marrow endothelial cells
Pdgfb	1.73E-43	2.00171617	0.642	0.043	9.57E-39	Bone marrow endothelial cells
Bvht	1.15E-42	1.53695804	0.632	0.088	6.39E-38	Bone marrow endothelial cells
Notch1	5.88E-42	1.65342243	0.802	0.134	3.26E-37	Bone marrow endothelial cells
Calm1	1.72E-41	1.64651688	0.953	0.735	9.53E-37	Bone marrow endothelial cells
S100a16	3.08E-41	1.73319556	0.858	0.268	1.71E-36	Bone marrow endothelial cells
Wwtr1	4.82E-41	1.64841529	0.792	0.238	2.67E-36	Bone marrow endothelial cells
Tfpi	5.28E-41	2.34355585	0.594	0.039	2.93E-36	Bone marrow endothelial cells
Ndrg1	7.14E-41	1.55945175	0.849	0.197	3.96E-36	Bone marrow endothelial cells
S1pr1	1.48E-40	1.77031003	0.877	0.238	8.18E-36	Bone marrow endothelial cells
Aplp2	2.46E-40	1.71991573	0.925	0.44	1.36E-35	Bone marrow endothelial cells
Bst2	2.95E-40	1.91001881	0.934	0.372	1.63E-35	Bone marrow endothelial cells
Actn4	3.20E-40	1.57273572	0.934	0.472	1.77E-35	Bone marrow endothelial cells
Nid1	4.01E-40	1.71009194	0.698	0.07	2.22E-35	Bone marrow endothelial cells
Trp53i11	5.52E-40	1.9019952	0.575	0.027	3.06E-35	Bone marrow endothelial cells
Adam15	8.49E-40	1.64030689	0.792	0.197	4.70E-35	Bone marrow endothelial cells
Tcn2	2.30E-39	1.89448239	0.821	0.447	1.27E-34	Bone marrow endothelial cells
Clic4	7.37E-39	1.83468485	0.858	0.342	4.09E-34	Bone marrow endothelial cells
Dusp3	8.63E-39	1.70278253	0.651	0.082	4.78E-34	Bone marrow endothelial cells
Tgfbr2	1.09E-38	1.58281464	0.821	0.243	6.06E-34	Bone marrow endothelial cells
Heg1	1.97E-38	1.82707129	0.726	0.186	1.09E-33	Bone marrow endothelial cells
Gimap6	5.59E-38	1.6948103	0.717	0.15	3.10E-33	Bone marrow endothelial cells
Efnb2	6.35E-38	1.69841996	0.557	0.027	3.52E-33	Bone marrow endothelial cells
Mafb	9.59E-38	2.82290123	0.755	0.231	5.31E-33	Bone marrow endothelial cells
Lrg1	3.26E-37	2.38065926	0.821	0.17	1.80E-32	Bone marrow endothelial cells
Lpar6	3.28E-37	1.66064717	0.66	0.077	1.82E-32	Bone marrow endothelial cells
Clec2d	4.51E-37	1.89318486	0.849	0.224	2.50E-32	Bone marrow endothelial cells
Cavin1	8.08E-37	1.71215071	0.83	0.24	4.48E-32	Bone marrow endothelial cells
Rbp1	3.67E-36	1.82213204	0.651	0.093	2.03E-31	Bone marrow endothelial cells
Ramp3	5.24E-36	1.75406708	0.472	0.009	2.90E-31	Bone marrow endothelial cells
Fcgrt	5.30E-36	1.82536625	0.877	0.433	2.94E-31	Bone marrow endothelial cells
Il6st	1.09E-35	2.54571997	0.783	0.27	6.05E-31	Bone marrow endothelial cells
Gpr182	1.09E-35	1.64045839	0.434	0.002	6.05E-31	Bone marrow endothelial cells
Igfbp3	2.38E-35	3.42759552	0.509	0.02	1.32E-30	Bone marrow endothelial cells
Txnip	5.54E-35	1.62303265	0.953	0.553	3.07E-30	Bone marrow endothelial cells
Rras	1.62E-34	1.5520348	0.726	0.172	8.98E-30	Bone marrow endothelial cells
Id1	1.02E-33	2.21495659	0.802	0.224	5.66E-29	Bone marrow endothelial cells
Nrp1	1.20E-33	1.50170436	0.792	0.204	6.66E-29	Bone marrow endothelial cells
Apold1	8.03E-33	1.75658464	0.5	0.032	4.45E-28	Bone marrow endothelial cells
Rhob	8.49E-32	1.7027247	0.925	0.515	4.71E-27	Bone marrow endothelial cells
AW112010	8.84E-32	1.88397423	0.632	0.079	4.90E-27	Bone marrow endothelial cells
Adamts1	1.24E-31	1.95653547	0.66	0.111	6.87E-27	Bone marrow endothelial cells
Ctsl	2.21E-31	2.41124869	0.896	0.601	1.23E-26	Bone marrow endothelial cells
Stab2	2.26E-31	1.938329	0.358	0	1.25E-26	Bone marrow endothelial cells
Fbln2	2.66E-31	2.47314828	0.443	0.029	1.47E-26	Bone marrow endothelial cells
Sema3g	7.29E-31	1.72746454	0.377	0.002	4.04E-26	Bone marrow endothelial cells

Bmpr2	1.03E-30	1.50146626	0.764	0.286	5.73E-26	Bone marrow endothelial cells
Aqp1	4.60E-30	2.35985177	0.679	0.243	2.55E-25	Bone marrow endothelial cells
Itga1	1.15E-29	1.59241373	0.632	0.095	6.35E-25	Bone marrow endothelial cells
Kcnj8	2.19E-29	1.86817299	0.443	0.02	1.21E-24	Bone marrow endothelial cells
F8	3.54E-29	1.79194861	0.415	0.025	1.96E-24	Bone marrow endothelial cells
Ednrb	5.62E-29	1.59244671	0.377	0.005	3.11E-24	Bone marrow endothelial cells
Col18a1	7.75E-29	1.5422576	0.481	0.032	4.29E-24	Bone marrow endothelial cells
Cyp4b1	9.23E-29	1.70959135	0.33	0	5.12E-24	Bone marrow endothelial cells
Nr2f2	1.51E-28	1.50405712	0.5	0.039	8.36E-24	Bone marrow endothelial cells
Tspan7	2.33E-28	1.80609588	0.491	0.059	1.29E-23	Bone marrow endothelial cells
Anxa3	3.29E-28	1.52971105	0.698	0.181	1.82E-23	Bone marrow endothelial cells
Gpm6a	6.68E-28	1.7335455	0.321	0	3.70E-23	Bone marrow endothelial cells
Pim3	1.92E-26	1.57726905	0.575	0.127	1.06E-21	Bone marrow endothelial cells
Depp1	3.62E-25	1.53590966	0.358	0.014	2.00E-20	Bone marrow endothelial cells
Plk2	4.83E-25	1.72028382	0.651	0.184	2.67E-20	Bone marrow endothelial cells
Mrc1	2.03E-24	1.77550816	0.434	0.032	1.13E-19	Bone marrow endothelial cells
Sgk1	2.55E-24	1.86976005	0.651	0.17	1.41E-19	Bone marrow endothelial cells
Jun	1.86E-23	1.87595295	0.943	0.569	1.03E-18	Bone marrow endothelial cells
Klf4	3.82E-23	1.6735097	0.83	0.367	2.11E-18	Bone marrow endothelial cells
Hspa1a	7.16E-23	2.53527313	0.708	0.229	3.97E-18	Bone marrow endothelial cells
Glul	1.29E-22	1.56431062	0.689	0.195	7.14E-18	Bone marrow endothelial cells
Hspa1b	1.59E-22	2.60452062	0.651	0.227	8.84E-18	Bone marrow endothelial cells
Npnt	1.47E-21	1.56117065	0.481	0.107	8.13E-17	Bone marrow endothelial cells
Rnase4	1.85E-21	1.51814988	0.528	0.213	1.02E-16	Bone marrow endothelial cells
Dusp6	2.07E-21	1.50270228	0.679	0.215	1.15E-16	Bone marrow endothelial cells
Sparcl1	2.84E-21	1.91849887	0.377	0.027	1.57E-16	Bone marrow endothelial cells
Jund	3.48E-21	1.54035757	0.991	0.794	1.93E-16	Bone marrow endothelial cells
Ubd	6.76E-21	1.95049048	0.264	0.002	3.75E-16	Bone marrow endothelial cells
Rsad2	1.70E-19	1.79824548	0.453	0.063	9.41E-15	Bone marrow endothelial cells
Fcgr2b	3.01E-19	2.04526798	0.368	0.059	1.67E-14	Bone marrow endothelial cells
Hes1	5.87E-19	1.52785759	0.642	0.22	3.25E-14	Bone marrow endothelial cells
7SK.293	1.41E-17	1.77853051	0.651	0.345	7.82E-13	Bone marrow endothelial cells
Gja4	1.54E-16	1.51862937	0.245	0.007	8.53E-12	Bone marrow endothelial cells
