# Supplementary material for: Single cell cortical bone transcriptomics define novel osteolineage gene sets altered in chronic kidney disease
Source: Front Endocrinol (Lausanne). 2023 Jan 26;14:1063083. doi: 10.3389/fendo.2023.1063083 (PMC9910177; doi:10.3389/fendo.2023.1063083)
Supplement: Supplementary file 2 [file DataSheet_2.pdf]

Up 3wkvsundiff (background allpeak)

Total Target Sequences = 8562, Total Background Sequences = 84141

Rank	Motif	Name	P-value	log P-pvalue	q-value (Benjamini)	# Target Sequences with Motif	% of Targets Sequences with Motif	# Background Sequences with Motif	% of Background Sequences with Motif
1	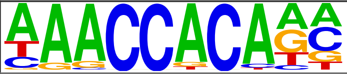	RUNX1(Runt)/Jurkat-RUNX1-ChIP-Seq(GSE29180)/Homer	1e-94	-2.186e+02	0.0000	4232.0	49.43%	32301.6	38.39%
2	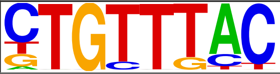	Foxo1(Forkhead)/RAW-Foxo1-ChIP-Seq(Fan_et_al.)/Homer	1e-79	-1.826e+02	0.0000	4786.0	55.90%	38446.1	45.69%
3	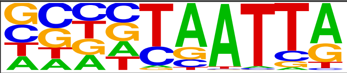	DLX1(Homeobox)/BasalGanglia-Dlx1-ChIP-seq(GSE124936)/Homer	1e-78	-1.809e+02	0.0000	3300.0	38.55%	24445.6	29.05%
4	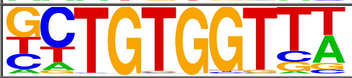	RUNX-AML(Runt)/CD4+-PolII-ChIP-Seq(Barski_et_al.)/Homer	1e-75	-1.741e+02	0.0000	3312.0	38.69%	24701.5	29.36%
5	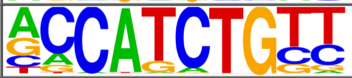	NeuroG2(bHLH)/Fibroblast-NeuroG2-ChIP-Seq(GSE75910)/Homer	1e-75	-1.733e+02	0.0000	4181.0	48.84%	32826.6	39.02%
6	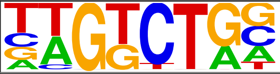	Smad3(MAD)/NPC-Smad3-ChIP-Seq(GSE36673)/Homer	1e-74	-1.719e+02	0.0000	6206.0	72.49%	53116.4	63.13%
7	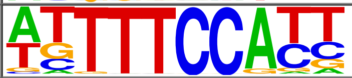	NFAT(RHD)/Jurkat-NFATC1-ChIP-Seq(Jolma_et_al.)/Homer	1e-72	-1.673e+02	0.0000	2699.0	31.53%	19341.8	22.99%
8	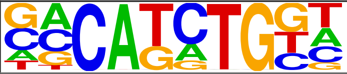	TCF4(bHLH)/SHSY5Y-TCF4-ChIP-Seq(GSE96915)/Homer	1e-71	-1.656e+02	0.0000	4159.0	48.58%	32802.7	38.99%
9	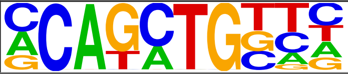	Twist2(bHLH)/Myoblast-Twist2.Ty1-ChIP-Seq(GSE127998)/Homer	1e-68	-1.582e+02	0.0000	4444.0	51.91%	35719.6	42.45%
10	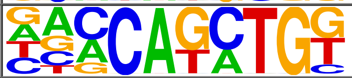	BHLHA15(bHLH)/NIH3T3-BHLHB8.HA-ChIP-Seq(GSE119782)/Homer	1e-68	-1.574e+02	0.0000	3863.0	45.12%	30197.8	35.89%
11	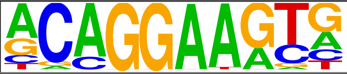	ERG(ETS)/VCaP-ERG-ChIP-Seq(GSE14097)/Homer	1e-68	-1.570e+02	0.0000	4370.0	51.05%	35037.7	41.64%
12	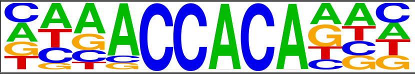	RUNX2(Runt)/PCa-RUNX2-ChIP-Seq(GSE33889)/Homer	1e-68	-1.568e+02	0.0000	3644.0	42.57%	28161.3	33.47%
13	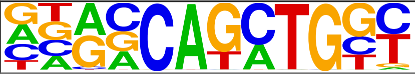	Atoh1(bHLH)/Cerebellum-Atoh1-ChIP-Seq(GSE22111)/Homer	1e-64	-1.475e+02	0.0000	3226.0	37.68%	24520.2	29.14%
14	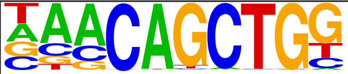	Tcf21(bHLH)/ArterySmoothMuscle-Tcf21-ChIP-Seq(GSE61369)/Homer	1e-63	-1.466e+02	0.0000	2939.0	34.33%	21931.5	26.07%
15	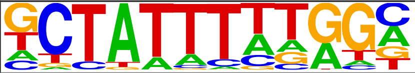	Mef2b(MADS)/HEK293-Mef2b.V5-ChIP-Seq(GSE67450)/Homer	1e-61	-1.419e+02	0.0000	2436.0	28.45%	17552.8	20.86%
16	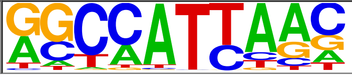	Nanog(Homeobox)/mES-Nanog-ChIP-Seq(GSE11724)/Homer	1e-60	-1.399e+02	0.0000	6855.0	80.07%	60873.4	72.35%

17	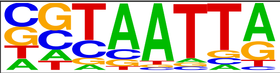	DLX5(Homeobox)/BasalGanglia-Dlx5-ChIP-seq(GSE124936)/Homer	1e-59	-1.380e+02	0.0000	2096.0	24.48%	14674.2	17.44%
18	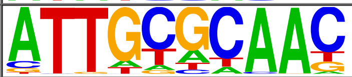	CEBP(bZIP)/ThioMac-CEBPb-ChIP-Seq(GSE21512)/Homer	1e-59	-1.379e+02	0.0000	2375.0	27.74%	17103.1	20.33%
19	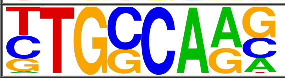	NF1-halfsite(CTF)/LNCaP-NF1-ChIP-Seq(Unpublished)/Homer	1e-59	-1.374e+02	0.0000	4755.0	55.54%	39299.3	46.71%
20	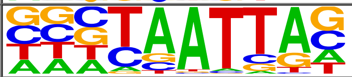	DLX2(Homeobox)/BasalGanglia-Dlx2-ChIP-seq(GSE124936)/Homer	1e-58	-1.357e+02	0.0000	3502.0	40.91%	27360.9	32.52%
21	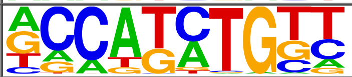	Olig2(bHLH)/Neuron-Olig2-ChIP-Seq(GSE30882)/Homer	1e-58	-1.349e+02	0.0000	4702.0	54.92%	38850.5	46.17%
22	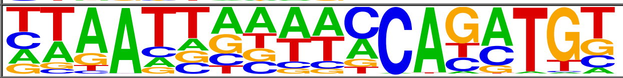	Pitx1:Ebox(Homeobox,bHLH)/Hindlimb-Pitx1-ChIP-Seq(GSE41591)/Homer	1e-58	-1.337e+02	0.0000	808.0	9.44%	4339.8	5.16%
23	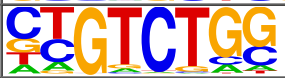	Smad2(MAD)/ES-SMAD2-ChIP-Seq(GSE29422)/Homer	1e-57	-1.331e+02	0.0000	4151.0	48.49%	33560.0	39.89%
24	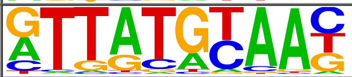	HLF(bZIP)/HSC-HLF.Flag-ChIP-Seq(GSE69817)/Homer	1e-57	-1.330e+02	0.0000	2502.0	29.23%	18330.7	21.79%
25	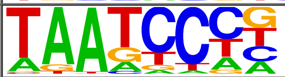	Pitx1(Homeobox)/Chicken-Pitx1-ChIP-Seq(GSE38910)/Homer	1e-57	-1.321e+02	0.0000	6876.0	80.32%	61304.9	72.86%
26	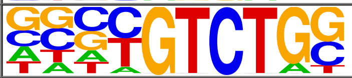	Smad4(MAD)/ESC-SMAD4-ChIP-Seq(GSE29422)/Homer	1e-55	-1.286e+02	0.0000	4184.0	48.87%	34002.7	40.41%
27	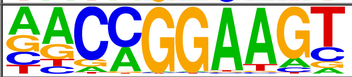	ETV1(ETS)/GIST48-ETV1-ChIP-Seq(GSE22441)/Homer	1e-54	-1.250e+02	0.0000	3825.0	44.68%	30679.3	36.46%
28	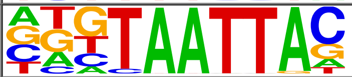	Dlx3(Homeobox)/Kerainocytes-Dlx3-ChIP-Seq(GSE89884)/Homer	1e-53	-1.241e+02	0.0000	1806.0	21.10%	12471.0	14.82%
29	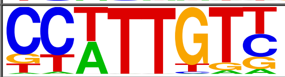	Sox3(HMG)/NPC-Sox3-ChIP-Seq(GSE33059)/Homer	1e-53	-1.241e+02	0.0000	4262.0	49.78%	34879.1	41.45%
30	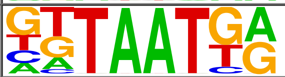	Nkx6.1(Homeobox)/Islet-Nkx6.1-ChIP-Seq(GSE40975)/Homer	1e-53	-1.239e+02	0.0000	4957.0	57.90%	41675.0	49.53%
31	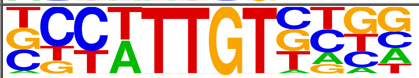	Sox21(HMG)/ESC-SOX21-ChIP-Seq(GSE110505)/Homer	1e-53	-1.230e+02	0.0000	4437.0	51.83%	36603.1	43.50%
32	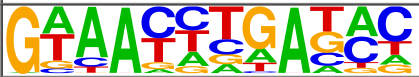	Six2(Homeobox)/NephronProgenitor-Six2-ChIP-Seq(GSE39837)/Homer	1e-53	-1.226e+02	0.0000	2972.0	34.72%	22809.7	27.11%
33	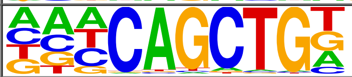	Ap4(bHLH)/AML-Tfap4-ChIP-Seq(GSE45738)/Homer	1e-53	-1.226e+02	0.0000	3466.0	40.49%	27370.6	32.53%
34	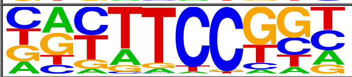	Fli1(ETS)/CD8-FLI-ChIP-Seq(GSE20898)/Homer	1e-52	-1.219e+02	0.0000	3291.0	38.44%	25760.0	30.62%
35	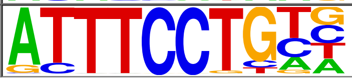	EWS:ERG-fusion(ETS)/CADO_ES1-EWS:ERG-ChIP-Seq(SRA014231)/Homer	1e-50	-1.168e+02	0.0000	2395.0	27.98%	17753.1	21.10%

36	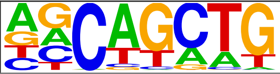	SCL(bHLH)/HPC7-Scl-ChIP-Seq(GSE13511)/Homer	1e-50	-1.163e+02	0.0000	7294.0	85.20%	66336.0	78.84%
37	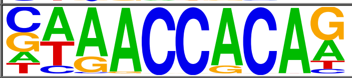	RUNX(Runt)/HPC7-Runx1-ChIP-Seq(GSE22178)/Homer	1e-50	-1.158e+02	0.0000	3240.0	37.85%	25451.4	30.25%
38	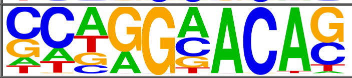	AR-halfsite(NR)/LNCaP-AR-ChIP-Seq(GSE27824)/Homer	1e-50	-1.156e+02	0.0000	6836.0	79.85%	61312.9	72.87%
39	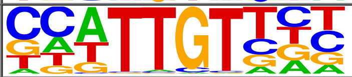	Sox6(HMG)/Myotubes-Sox6-ChIP-Seq(GSE32627)/Homer	1e-50	-1.155e+02	0.0000	3962.0	46.28%	32249.8	38.33%
40	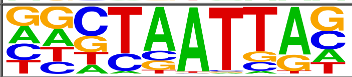	En1(Homeobox)/SUM149-EN1-ChIP-Seq(GSE120957)/Homer	1e-49	-1.134e+02	0.0000	4019.0	46.95%	32855.5	39.05%
41	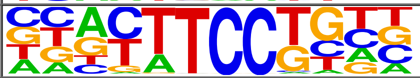	Etv2(ETS)/ES-ER71-ChIP-Seq(GSE59402)/Homer	1e-47	-1.084e+02	0.0000	2949.0	34.45%	22971.2	27.30%
42	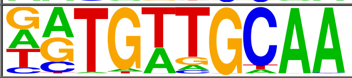	CEBP:AP1(bZIP)/ThioMac-CEBPb-ChIP-Seq(GSE21512)/Homer	1e-47	-1.083e+02	0.0000	2324.0	27.15%	17327.1	20.59%
43	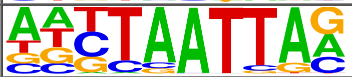	Lhx3(Homeobox)/Neuron-Lhx3-ChIP-Seq(GSE31456)/Homer	1e-46	-1.075e+02	0.0000	3616.0	42.24%	29203.8	34.71%
44	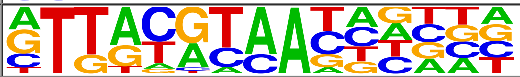	NFIL3(bZIP)/HepG2-NFIL3-ChIP-Seq(Encode)/Homer	1e-46	-1.072e+02	0.0000	2016.0	23.55%	14639.2	17.40%
45	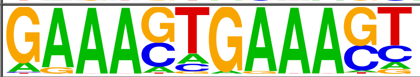	IRF1(IRF)/PBMC-IRF1-ChIP-Seq(GSE43036)/Homer	1e-46	-1.071e+02	0.0000	709.0	8.28%	3920.1	4.66%
46	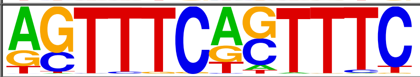	ISRE(IRF)/ThioMac-LPS-Expression(GSE23622)/Homer	1e-46	-1.068e+02	0.0000	408.0	4.77%	1812.1	2.15%
47	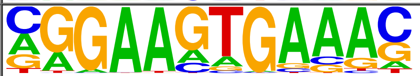	PU.1-IRF(ETS:IRF)/Bcell-PU.1-ChIP-Seq(GSE21512)/Homer	1e-46	-1.067e+02	0.0000	3597.0	42.02%	29046.2	34.52%
48	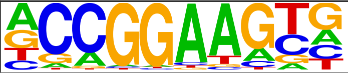	ETV4(ETS)/HepG2-ETV4-ChIP-Seq(ENCODE)/Homer	1e-46	-1.063e+02	0.0000	3197.0	37.34%	25318.1	30.09%
49	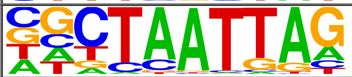	LHX9(Homeobox)/Hct116-LHX9.V5-ChIP-Seq(GSE116822)/Homer	1e-45	-1.056e+02	0.0000	3264.0	38.13%	25959.7	30.85%
50	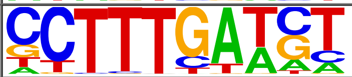	LEF1(HMG)/H1-LEF1-ChIP-Seq(GSE64758)/Homer	1e-45	-1.053e+02	0.0000	1989.0	23.23%	14448.5	17.17%
51	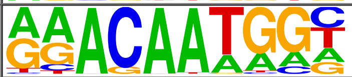	Sox15(HMG)/CPA-Sox15-ChIP-Seq(GSE62909)/Homer	1e-45	-1.052e+02	0.0000	2928.0	34.20%	22867.6	27.18%
52	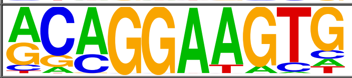	ETS1(ETS)/Jurkat-ETS1-ChIP-Seq(GSE17954)/Homer	1e-45	-1.049e+02	0.0000	3151.0	36.81%	24931.2	29.63%
53	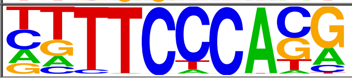	Rbpj1(?)/Panc1-Rbpj1-ChIP-Seq(GSE47459)/Homer	1e-45	-1.045e+02	0.0000	3888.0	45.42%	31871.5	37.88%

54	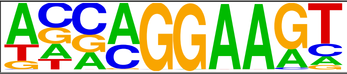	EHF(ETS)/LoVo-EHF-ChIP-Seq(GSE49402)/Homer	1e-44	-1.023e+02	0.0000	3538.0	41.33%	28622.6	34.02%
55	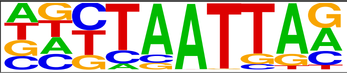	Lhx1(Homeobox)/EmbryoCarcinoma-Lhx1-ChIP-Seq(GSE70957)/Homer	1e-44	-1.018e+02	0.0000	2581.0	30.15%	19797.3	23.53%
56	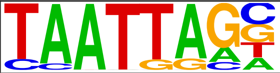	Lhx2(Homeobox)/HFSC-Lhx2-ChIP-Seq(GSE48068)/Homer	1e-44	-1.015e+02	0.0000	2545.0	29.73%	19480.5	23.15%
57	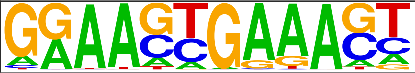	IRF8(IRF)/BMDM-IRF8-ChIP-Seq(GSE77884)/Homer	1e-43	-1.009e+02	0.0000	1234.0	14.41%	8132.1	9.67%
58	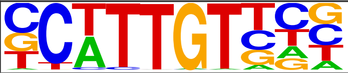	Sox10(HMG)/SciaticNerve-Sox3-ChIP-Seq(GSE35132)/Homer	1e-43	-1.007e+02	0.0000	4077.0	47.62%	33799.6	40.17%
59	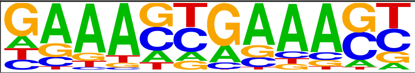	IRF2(IRF)/Erythroblas-IRF2-ChIP-Seq(GSE36985)/Homer	1e-43	-9.948e+01	0.0000	590.0	6.89%	3137.6	3.73%
60	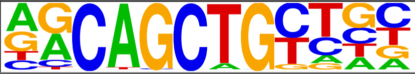	MyoD(bHLH)/Myotube-MyoD-ChIP-Seq(GSE21614)/Homer	1e-42	-9.857e+01	0.0000	2370.0	27.68%	17983.9	21.37%
61	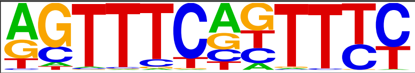	IRF3(IRF)/BMDM-Irf3-ChIP-Seq(GSE67343)/Homer	1e-41	-9.635e+01	0.0000	1299.0	15.17%	8754.0	10.40%
62	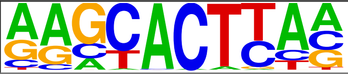	Nkx3.1(Homeobox)/LNCaP-Nkx3.1-ChIP-Seq(GSE28264)/Homer	1e-39	-9.207e+01	0.0000	5035.0	58.81%	43451.3	51.64%
63	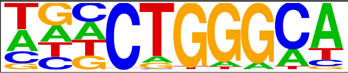	ZNF416(Zf)/HEK293-ZNF416.GFP-ChIP-Seq(GSE58341)/Homer	1e-39	-9.198e+01	0.0000	3662.0	42.78%	30115.4	35.79%
64	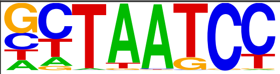	CRX(Homeobox)/Retina-Crx-ChIP-Seq(GSE20012)/Homer	1e-39	-9.150e+01	0.0000	4793.0	55.99%	41074.7	48.82%
65	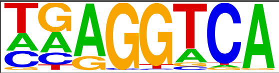	THRb(NR)/Liver-NR1A2-ChIP-Seq(GSE52613)/Homer	1e-39	-9.115e+01	0.0000	6763.0	79.00%	61245.7	72.79%
66	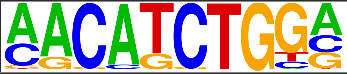	ZBTB18(Zf)/HEK293-ZBTB18.GFP-ChIP-Seq(GSE58341)/Homer	1e-39	-9.049e+01	0.0000	1697.0	19.82%	12253.4	14.56%
67	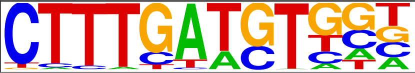	Tcf7(HMG)/GM12878-TCF7-ChIP-Seq(Encode)/Homer	1e-38	-8.872e+01	0.0000	1141.0	13.33%	7593.0	9.02%
68	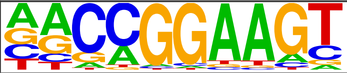	GABPA(ETS)/Jurkat-GABPa-ChIP-Seq(GSE17954)/Homer	1e-38	-8.821e+01	0.0000	2764.0	32.29%	21847.5	25.97%
69	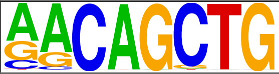	MyoG(bHLH)/C2C12-MyoG-ChIP-Seq(GSE36024)/Homer	1e-37	-8.707e+01	0.0000	2830.0	33.06%	22488.6	26.73%
70	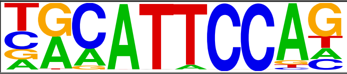	TEAD3(TEA)/HepG2-TEAD3-ChIP-Seq(Encode)/Homer	1e-37	-8.566e+01	0.0000	3469.0	40.52%	28494.7	33.87%
71	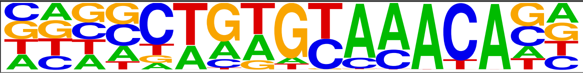	Fox:Ebox(Forkhead,bHLH)/Panc1-Foxa2-ChIP-Seq(GSE47459)/Homer	1e-37	-8.556e+01	0.0000	3006.0	35.11%	24163.0	28.72%
72	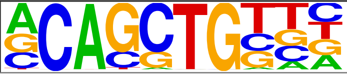	Ptf1a(bHLH)/Panc1-Ptf1a-ChIP-Seq(GSE47459)/Homer	1e-36	-8.400e+01	0.0000	5058.0	59.08%	43961.7	52.25%

73	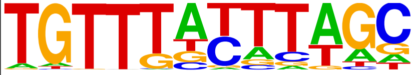	FoxD3(forkhead)/ZebrafishEmbryo-Foxd3.biotin-ChIP-seq(GSE106676)/Homer	1e-36	-8.396e+01	0.0000	2787.0	32.55%	22187.4	26.37%
74	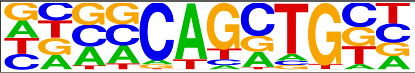	Ascl1(bHLH)/NeuralTubes-Ascl1-ChIP-Seq(GSE55840)/Homer	1e-36	-8.369e+01	0.0000	3655.0	42.69%	30322.5	36.04%
75	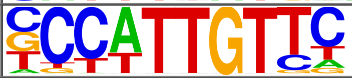	Sox2(HMG)/mES-Sox2-ChIP-Seq(GSE11431)/Homer	1e-36	-8.363e+01	0.0000	2479.0	28.96%	19379.7	23.03%
76	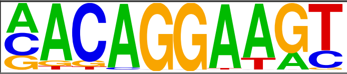	Ets1-distal(ETS)/CD4+-PolII-ChIP-Seq(Barski_et_al.)/Homer	1e-36	-8.347e+01	0.0000	1236.0	14.44%	8482.3	10.08%
77	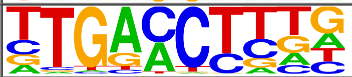	RARa(NR)/K562-RARa-ChIP-Seq(Encode)/Homer	1e-36	-8.331e+01	0.0000	5622.0	65.67%	49652.2	59.01%
78	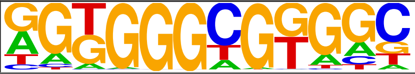	KLF14(Zf)/HEK293-KLF14.GFP-ChIP-Seq(GSE58341)/Homer	1e-36	-8.320e+01	0.0000	3922.0	45.81%	32889.7	39.09%
79	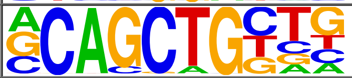	Tcf12(bHLH)/GM12878-Tcf12-ChIP-Seq(GSE32465)/Homer	1e-36	-8.290e+01	0.0000	2730.0	31.89%	21695.7	25.79%
80	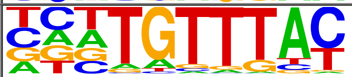	FOXK1(Forkhead)/HEK293-FOXK1-ChIP-Seq(GSE51673)/Homer	1e-35	-8.275e+01	0.0000	2823.0	32.98%	22556.0	26.81%
81	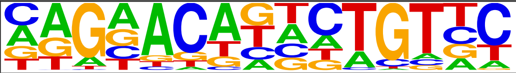	PR(NR)/T47D-PR-ChIP-Seq(GSE31130)/Homer	1e-35	-8.141e+01	0.0000	5030.0	58.75%	43776.7	52.03%
82	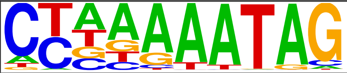	Mef2a(MADS)/HL1-Mef2a.biotin-ChIP-Seq(GSE21529)/Homer	1e-35	-8.139e+01	0.0000	1316.0	15.37%	9198.3	10.93%
83	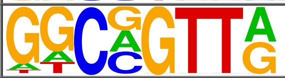	MYB(HTH)/ERMYB-Myb-ChIPSeq(GSE22095)/Homer	1e-35	-8.114e+01	0.0000	4139.0	48.35%	35051.5	41.66%
84	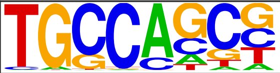	HIC1(Zf)/Treg-ZBTB29-ChIP-Seq(GSE99889)/Homer	1e-35	-8.075e+01	0.0000	4780.0	55.83%	41324.8	49.12%
85	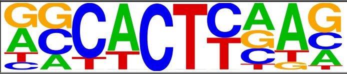	Nkx2.1(Homeobox)/LungAC-Nkx2.1-ChIP-Seq(GSE43252)/Homer	1e-35	-8.071e+01	0.0000	5765.0	67.34%	51199.5	60.85%
86	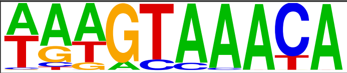	FOXA1(Forkhead)/LNCAP-FOXA1-ChIP-Seq(GSE27824)/Homer	1e-35	-8.060e+01	0.0000	3289.0	38.42%	26966.2	32.05%
87	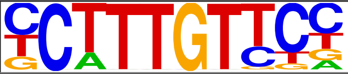	Sox4(HMG)/proB-Sox4-ChIP-Seq(GSE50066)/Homer	1e-34	-7.982e+01	0.0000	2450.0	28.62%	19228.3	22.85%
88	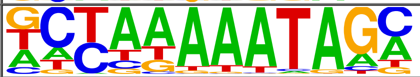	Mef2c(MADS)/GM12878-Mef2c-ChIP-Seq(GSE32465)/Homer	1e-34	-7.959e+01	0.0000	1406.0	16.42%	10002.6	11.89%
89	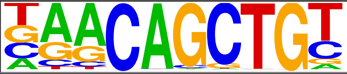	Myf5(bHLH)/GM-Myf5-ChIP-Seq(GSE24852)/Homer	1e-34	-7.953e+01	0.0000	2086.0	24.37%	15954.5	18.96%
90	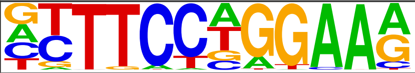	STAT4(Stat)/CD4-Stat4-ChIP-Seq(GSE22104)/Homer	1e-33	-7.763e+01	0.0000	2785.0	32.53%	22367.0	26.58%

91	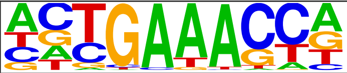	IRF4(IRF)/GM12878-IRF4-ChIP-Seq(GSE32465)/Homer	1e-32	-7.427e+01	0.0000	1427.0	16.67%	10305.8	12.25%
92	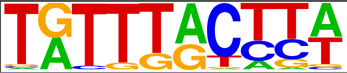	FOXM1(Forkhead)/MCF7-FOXM1-ChIP-Seq(GSE72977)/Homer	1e-32	-7.420e+01	0.0000	2951.0	34.47%	24019.6	28.55%
93	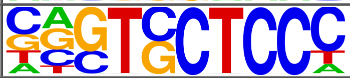	Znf263(Zf)/K562-Znf263-ChIP-Seq(GSE31477)/Homer	1e-32	-7.385e+01	0.0000	4056.0	47.38%	34514.4	41.02%
94	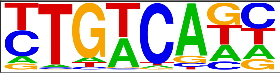	Tgif1(Homeobox)/mES-Tgif1-ChIP-Seq(GSE55404)/Homer	1e-32	-7.371e+01	0.0000	5823.0	68.02%	52047.3	61.86%
95	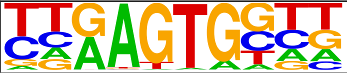	Bapx1(Homeobox)/VertebralCol-Bapx1-ChIP-Seq(GSE36672)/Homer	1e-31	-7.365e+01	0.0000	4707.0	54.98%	40869.1	48.57%
96	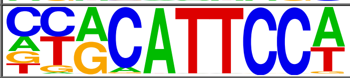	TEAD1(TEAD)/HepG2-TEAD1-ChIP-Seq(Encode)/Homer	1e-31	-7.360e+01	0.0000	3107.0	36.29%	25496.3	30.30%
97	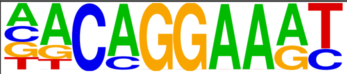	EWS:FLI1-fusion(ETS)/SK_N_MC-EWS:FLI1-ChIP-Seq(SRA014231)/Homer	1e-31	-7.333e+01	0.0000	1947.0	22.74%	14887.7	17.69%
98	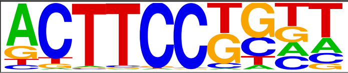	Elf4(ETS)/BMDM-Elf4-ChIP-Seq(GSE88699)/Homer	1e-31	-7.205e+01	0.0000	2916.0	34.06%	23766.4	28.25%
99	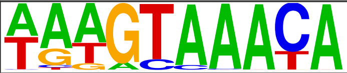	FOXA1(Forkhead)/MCF7-FOXA1-ChIP-Seq(GSE26831)/Homer	1e-31	-7.146e+01	0.0000	2842.0	33.20%	23098.2	27.45%
100	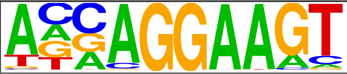	ELF3(ETS)/PDAC-ELF3-ChIP-Seq(GSE64557)/Homer	1e-30	-7.113e+01	0.0000	2255.0	26.34%	17721.9	21.06%
101	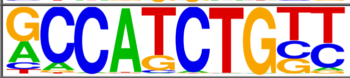	NeuroD1(bHLH)/Islet-NeuroD1-ChIP-Seq(GSE30298)/Homer	1e-30	-7.108e+01	0.0000	2428.0	28.36%	19297.7	22.94%
102	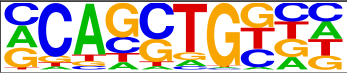	HEB(bHLH)/mES-Heb-ChIP-Seq(GSE53233)/Homer	1e-30	-7.017e+01	0.0000	3857.0	45.05%	32736.5	38.91%
103	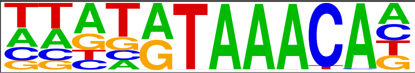	Foxf1(Forkhead)/Lung-Foxf1-ChIP-Seq(GSE77951)/Homer	1e-29	-6.889e+01	0.0000	2528.0	29.53%	20284.7	24.11%
104	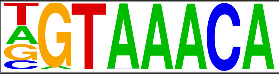	Foxo3(Forkhead)/U2OS-Foxo3-ChIP-Seq(E-MTAB-2701)/Homer	1e-29	-6.864e+01	0.0000	2191.0	25.59%	17218.3	20.46%
105	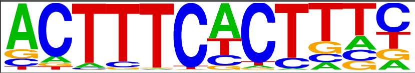	PRDM1(Zf)/Hela-PRDM1-ChIP-Seq(GSE31477)/Homer	1e-29	-6.849e+01	0.0000	1671.0	19.52%	12576.3	14.95%
106	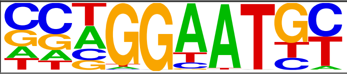	TEAD4(TEA)/Tropoblast-Tead4-ChIP-Seq(GSE37350)/Homer	1e-29	-6.810e+01	0.0000	2789.0	32.58%	22721.2	27.00%
107	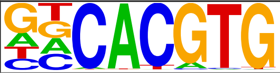	BMAL1(bHLH)/Liver-Bmal1-ChIP-Seq(GSE39860)/Homer	1e-29	-6.749e+01	0.0000	3939.0	46.01%	33627.8	39.97%
108	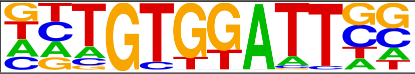	Foxh1(Forkhead)/hESC-FOXH1-ChIP-Seq(GSE29422)/Homer	1e-29	-6.727e+01	0.0000	1720.0	20.09%	13042.3	15.50%
109	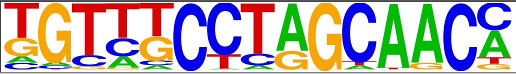	Rfx6(HTH)/Min6b1-Rfx6.HA-ChIP-Seq(GSE62844)/Homer	1e-29	-6.719e+01	0.0000	2964.0	34.62%	24383.9	28.98%

110	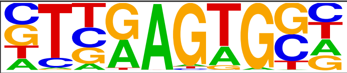	Nkx2.2(Homeobox)/NPC-Nkx2.2-ChIP-Seq(GSE61673)/Homer	1e-28	-6.660e+01	0.0000	4424.0	51.68%	38368.5	45.60%
111	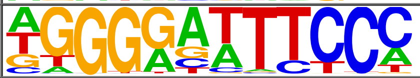	NFkB-p65(RHD)/GM12787-p65-ChIP-Seq(GSE19485)/Homer	1e-28	-6.632e+01	0.0000	1551.0	18.12%	11581.6	13.77%
112	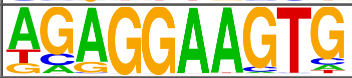	PU.1(ETS)/ThioMac-PU.1-ChIP-Seq(GSE21512)/Homer	1e-28	-6.494e+01	0.0000	1654.0	19.32%	12524.3	14.89%
113	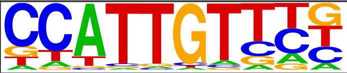	Sox17(HMG)/Endoderm-Sox17-ChIP-Seq(GSE61475)/Homer	1e-28	-6.452e+01	0.0000	1995.0	23.30%	15576.2	18.51%
114	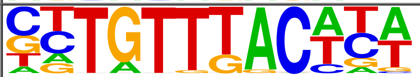	Foxa2(Forkhead)/Liver-Foxa2-ChIP-Seq(GSE25694)/Homer	1e-28	-6.449e+01	0.0000	2248.0	26.26%	17867.0	21.24%
115	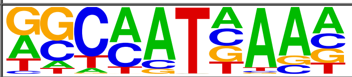	Hoxa9(Homeobox)/ChickenMSG-Hoxa9.Flag-ChIP-Seq(GSE86088)/Homer	1e-27	-6.389e+01	0.0000	5209.0	60.85%	46235.1	54.95%
116	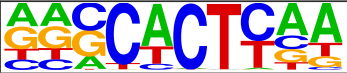	Nkx2.5(Homeobox)/HL1-Nkx2.5.biotin-ChIP-Seq(GSE21529)/Homer	1e-27	-6.374e+01	0.0000	4832.0	56.44%	42494.5	50.51%
117	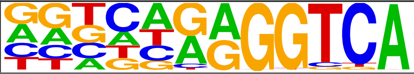	EAR2(NR)/K562-NR2F6-ChIP-Seq(Encode)/Homer	1e-27	-6.360e+01	0.0000	3231.0	37.74%	27023.1	32.12%
118	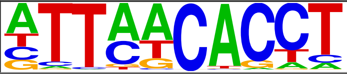	Eomes(T-box)/H9-Eomes-ChIP-Seq(GSE26097)/Homer	1e-27	-6.282e+01	0.0000	4190.0	48.94%	36241.3	43.07%
119	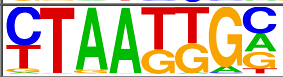	Isl1(Homeobox)/Neuron-Isl1-ChIP-Seq(GSE31456)/Homer	1e-26	-6.204e+01	0.0000	4119.0	48.11%	35584.2	42.29%
120	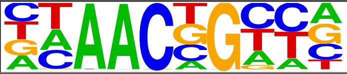	BMYB(HTH)/Hela-BMYB-ChIP-Seq(GSE27030)/Homer	1e-26	-6.186e+01	0.0000	3716.0	43.41%	31706.8	37.68%
121	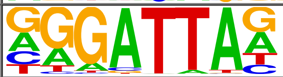	GSC(Homeobox)/FrogEmbryos-GSC-ChIP-Seq(DRA000576)/Homer	1e-26	-6.170e+01	0.0000	2709.0	31.64%	22204.6	26.39%
122	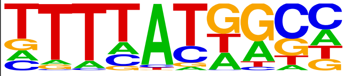	Hoxa11(Homeobox)/ChickenMSG-Hoxa11.Flag-ChIP-Seq(GSE86088)/Homer	1e-26	-6.158e+01	0.0000	4739.0	55.36%	41665.6	49.52%
123	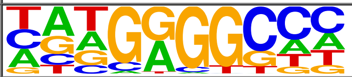	Zac1(Zf)/Neuro2A-Plagl1-ChIP-Seq(GSE75942)/Homer	1e-26	-6.145e+01	0.0000	5108.0	59.67%	45328.7	53.87%
124	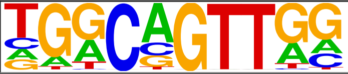	AMYB(HTH)/Testes-AMYB-ChIP-Seq(GSE44588)/Homer	1e-26	-6.124e+01	0.0000	3798.0	44.36%	32518.8	38.65%
125	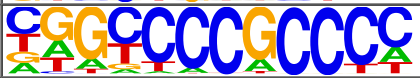	Sp2(Zf)/HEK293-Sp2.eGFP-ChIP-Seq(Encode)/Homer	1e-26	-6.112e+01	0.0000	3536.0	41.30%	30014.1	35.67%
126	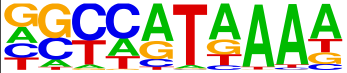	Hoxd11(Homeobox)/ChickenMSG-Hoxd11.Flag-ChIP-Seq(GSE86088)/Homer	1e-26	-6.088e+01	0.0000	4841.0	56.55%	42702.2	50.75%
127	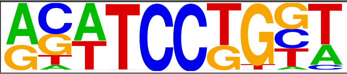	SPDEF(ETS)/VCaP-SPDEF-ChIP-Seq(SRA014231)/Homer	1e-26	-6.069e+01	0.0000	2896.0	33.83%	23984.5	28.51%
128	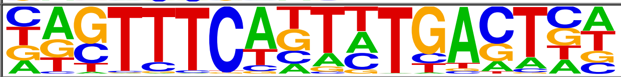	bZIP:IRF(bZIP,IRF)/Th17-BatF-ChIP-Seq(GSE39756)/Homer	1e-26	-6.013e+01	0.0000	1415.0	16.53%	10558.8	12.55%

129	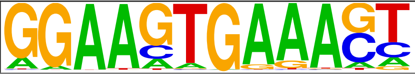	PU.1:IRF8(ETS:IRF)/pDC-Irf8-ChIP-Seq(GSE66899)/Homer	1e-26	-5.996e+01	0.0000	736.0	8.60%	4823.6	5.73%
130	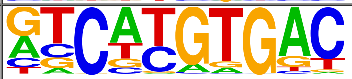	MITF(bHLH)/MastCells-MITF-ChIP-Seq(GSE48085)/Homer	1e-25	-5.863e+01	0.0000	2589.0	30.24%	21202.6	25.20%
131	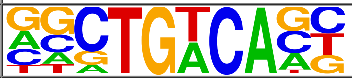	Meis1(Homeobox)/MastCells-Meis1-ChIP-Seq(GSE48085)/Homer	1e-25	-5.797e+01	0.0000	3939.0	46.01%	34011.5	40.42%
132	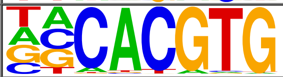	NPAS(bHLH)/Liver-NPAS-ChIP-Seq(GSE39860)/Homer	1e-25	-5.775e+01	0.0000	3593.0	41.97%	30695.1	36.48%
133	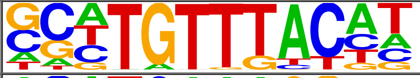	FOXK2(Forkhead)/U2OS-FOXK2-ChIP-Seq(E-MTAB-2204)/Homer	1e-24	-5.753e+01	0.0000	1871.0	21.85%	14679.6	17.45%
134	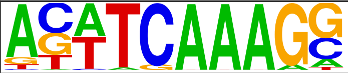	Tcf3(HMG)/mES-Tcf3-ChIP-Seq(GSE11724)/Homer	1e-24	-5.688e+01	0.0000	836.0	9.77%	5701.5	6.78%
135	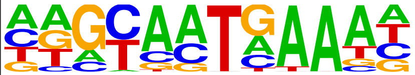	Hoxd12(Homeobox)/ChickenMSG-Hoxd12.Flag-ChIP-Seq(GSE86088)/Homer	1e-24	-5.669e+01	0.0000	3874.0	45.25%	33438.1	39.74%
136	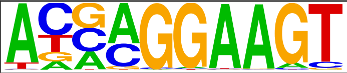	ELF5(ETS)/T47D-ELF5-ChIP-Seq(GSE30407)/Homer	1e-24	-5.549e+01	0.0000	2173.0	25.38%	17486.4	20.78%
137	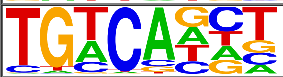	Tgif2(Homeobox)/mES-Tgif2-ChIP-Seq(GSE55404)/Homer	1e-24	-5.544e+01	0.0000	6085.0	71.08%	55454.0	65.91%
138	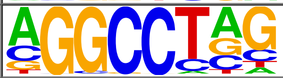	ZNF711(Zf)/SHSY5Y-ZNF711-ChIP-Seq(GSE20673)/Homer	1e-23	-5.485e+01	0.0000	3759.0	43.91%	32409.6	38.52%
139	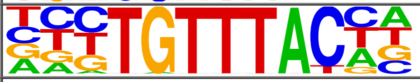	FOXP1(Forkhead)/H9-FOXP1-ChIP-Seq(GSE31006)/Homer	1e-23	-5.473e+01	0.0000	1381.0	16.13%	10409.8	12.37%
140	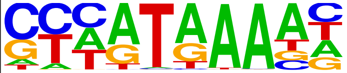	Hoxa13(Homeobox)/ChickenMSG-Hoxa13.Flag-ChIP-Seq(GSE86088)/Homer	1e-23	-5.462e+01	0.0000	4915.0	57.41%	43705.0	51.94%
141	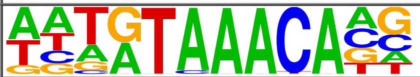	FoxL2(Forkhead)/Ovary-FoxL2-ChIP-Seq(GSE60858)/Homer	1e-23	-5.445e+01	0.0000	2356.0	27.52%	19202.0	22.82%
142	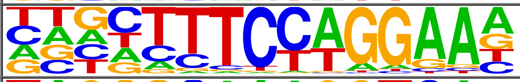	Bcl6(Zf)/Liver-Bcl6-ChIP-Seq(GSE31578)/Homer	1e-23	-5.370e+01	0.0000	3598.0	42.03%	30913.3	36.74%
143	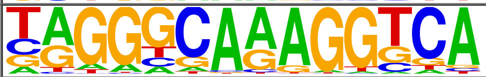	RXR(NR),DR1/3T3L1-RXR-ChIP-Seq(GSE13511)/Homer	1e-23	-5.358e+01	0.0000	2868.0	33.50%	23996.2	28.52%
144	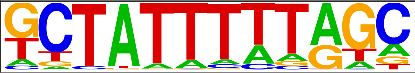	Mef2d(MADS)/Retina-Mef2d-ChIP-Seq(GSE61391)/Homer	1e-23	-5.323e+01	0.0000	716.0	8.36%	4788.6	5.69%
145	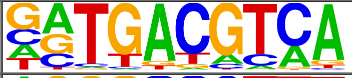	Atf1(bZIP)/K562-ATF1-ChIP-Seq(GSE31477)/Homer	1e-22	-5.216e+01	0.0000	1782.0	20.82%	14052.3	16.70%
146	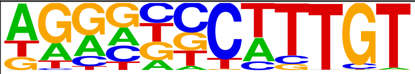	Sox9(HMG)/Limb-SOX9-ChIP-Seq(GSE73225)/Homer	1e-21	-5.007e+01	0.0000	2472.0	28.88%	20440.4	24.29%

147	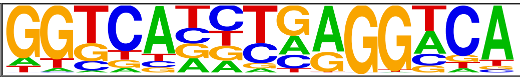	THRa(NR)/C17.2-THRa-ChIP-Seq(GSE38347)/Homer	1e-21	-4.932e+01	0.0000	1236.0	14.44%	9293.8	11.05%
148	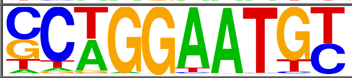	TEAD2(TEA)/Py2T-Tead2-ChIP-Seq(GSE55709)/Homer	1e-21	-4.857e+01	0.0000	1878.0	21.94%	15042.2	17.88%
149	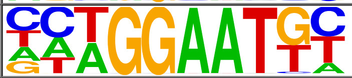	TEAD(TEA)/Fibroblast-PU.1-ChIP-Seq(Unpublished)/Homer	1e-20	-4.792e+01	0.0000	2281.0	26.64%	18756.2	22.29%
150	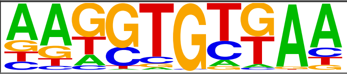	Tbr1(T-box)/Cortex-Tbr1-ChIP-Seq(GSE71384)/Homer	1e-20	-4.784e+01	0.0000	3047.0	35.59%	25921.0	30.81%
151	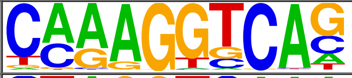	Erra(NR)/HepG2-Erra-ChIP-Seq(GSE31477)/Homer	1e-20	-4.648e+01	0.0000	4795.0	56.01%	42896.0	50.98%
152	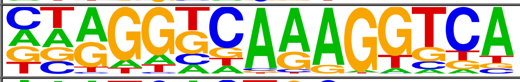	PPARa(NR),DR1/Liver-Ppara-ChIP-Seq(GSE47954)/Homer	1e-20	-4.647e+01	0.0000	2717.0	31.74%	22872.8	27.18%
153	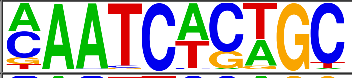	Gfi1b(Zf)/HPC7-Gfi1b-ChIP-Seq(GSE22178)/Homer	1e-19	-4.533e+01	0.0000	1873.0	21.88%	15113.6	17.96%
154	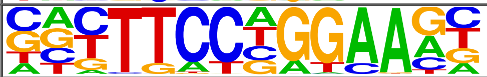	Stat3+il21(Stat)/CD4-Stat3-ChIP-Seq(GSE19198)/Homer	1e-19	-4.525e+01	0.0000	2135.0	24.94%	17514.8	20.82%
155	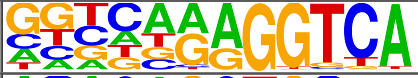	COUP-TFII(NR)/K562-NR2F1-ChIP-Seq(Encode)/Homer	1e-19	-4.500e+01	0.0000	3422.0	39.97%	29616.9	35.20%
156	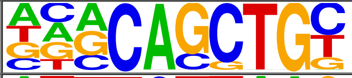	E2A(bHLH)/proBcell-E2A-ChIP-Seq(GSE21978)/Homer	1e-19	-4.484e+01	0.0000	2946.0	34.41%	25098.8	29.83%
157	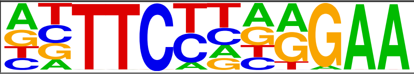	STAT6(Stat)/CD4-Stat6-ChIP-Seq(GSE22104)/Homer	1e-19	-4.400e+01	0.0000	1588.0	18.55%	12581.5	14.95%
158	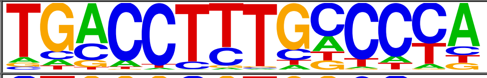	PPARE(NR),DR1/3T3L1-Pparg-ChIP-Seq(GSE13511)/Homer	1e-19	-4.399e+01	0.0000	2480.0	28.97%	20761.2	24.68%
159	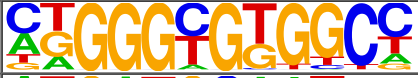	KLF6(Zf)/PDAC-KLF6-ChIP-Seq(GSE64557)/Homer	1e-18	-4.294e+01	0.0000	2770.0	32.36%	23523.1	27.96%
160	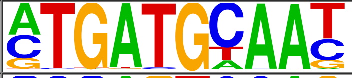	Atf4(bZIP)/MEF-Atf4-ChIP-Seq(GSE35681)/Homer	1e-18	-4.228e+01	0.0000	909.0	10.62%	6654.3	7.91%
161	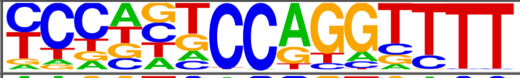	PRDM15(Zf)/ESC-Prdm15-ChIP-Seq(GSE73694)/Homer	1e-17	-4.141e+01	0.0000	2675.0	31.25%	22698.0	26.98%
162	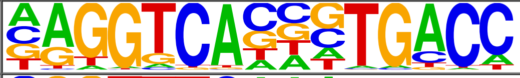	ERE(NR),IR3/MCF7-ERa-ChIP-Seq(Unpublished)/Homer	1e-17	-4.123e+01	0.0000	873.0	10.20%	6373.7	7.58%
163	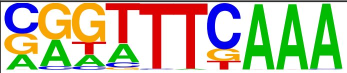	CHR(?)/Hela-CellCycle-Expression/Homer	1e-17	-4.002e+01	0.0000	1680.0	19.62%	13555.3	16.11%
164	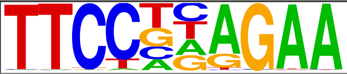	STAT6(Stat)/Macrophage-Stat6-ChIP-Seq(GSE38377)/Homer	1e-17	-3.988e+01	0.0000	1630.0	19.04%	13107.2	15.58%
165	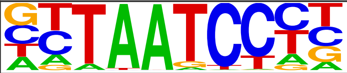	Otx2(Homeobox)/EpiLC-Otx2-ChIP-Seq(GSE56098)/Homer	1e-17	-3.952e+01	0.0000	1921.0	22.44%	15774.5	18.75%

166	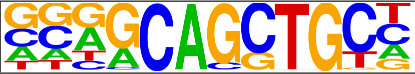	Ascl2(bHLH)/ESC-Ascl2-ChIP-Seq(GSE97712)/Homer	1e-17	-3.926e+01	0.0000	2442.0	28.52%	20611.2	24.50%
167	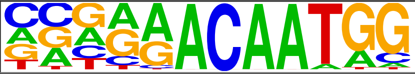	Sox7(HMG)/ESC-Sox7-ChIP-Seq(GSE133899)/Homer	1e-16	-3.911e+01	0.0000	893.0	10.43%	6601.1	7.85%
168	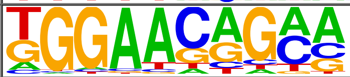	ZNF189(Zf)/HEK293-ZNF189.GFP-ChIP-Seq(GSE58341)/Homer	1e-16	-3.826e+01	0.0000	2291.0	26.76%	19247.3	22.88%
169	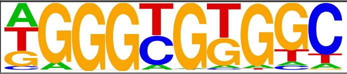	KLF5(Zf)/LoVo-KLF5-ChIP-Seq(GSE49402)/Homer	1e-16	-3.789e+01	0.0000	3188.0	37.24%	27723.3	32.95%
170	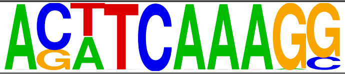	TCFL2(HMG)/K562-TCF7L2-ChIP-Seq(GSE29196)/Homer	1e-16	-3.776e+01	0.0000	320.0	3.74%	1910.5	2.27%
171	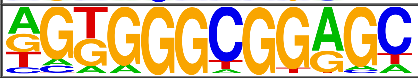	Sp5(Zf)/mES-Sp5.Flag-ChIP-Seq(GSE72989)/Homer	1e-16	-3.731e+01	0.0000	2587.0	30.22%	22059.2	26.22%
172	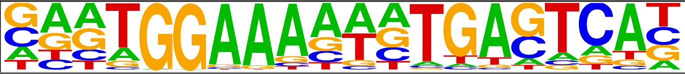	NFAT:AP1(RHD,bZIP)/Jurkat-NFATC1-ChIP-Seq(Jolma_et_al.)/Homer	1e-16	-3.730e+01	0.0000	661.0	7.72%	4673.9	5.56%
173	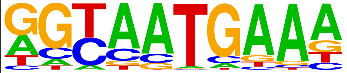	Hoxa10(Homeobox)/ChickenMSG-Hoxa10.Flag-ChIP-Seq(GSE86088)/Homer	1e-15	-3.572e+01	0.0000	1489.0	17.39%	11986.0	14.25%
174	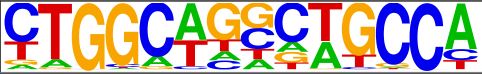	Tlx?(NR)/NPC-H3K4me1-ChIP-Seq(GSE16256)/Homer	1e-15	-3.567e+01	0.0000	1562.0	18.25%	12649.4	15.03%
175	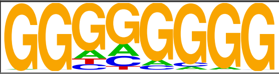	Maz(Zf)/HepG2-Maz-ChIP-Seq(GSE31477)/Homer	1e-15	-3.535e+01	0.0000	2903.0	33.91%	25139.2	29.88%
176	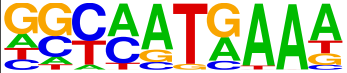	Hoxd10(Homeobox)/ChickenMSG-Hoxd10.Flag-ChIP-Seq(GSE86088)/Homer	1e-15	-3.483e+01	0.0000	2717.0	31.74%	23404.6	27.82%
177	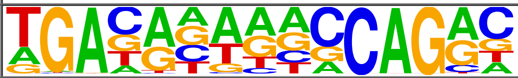	Hand2(bHLH)/Mesoderm-Hand2-ChIP-Seq(GSE61475)/Homer	1e-14	-3.425e+01	0.0000	1428.0	16.68%	11490.6	13.66%
178	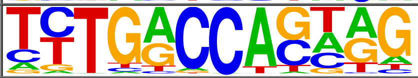	Bcl11a(Zf)/HSPC-BCL11A-ChIP-Seq(GSE104676)/Homer	1e-14	-3.419e+01	0.0000	2258.0	26.38%	19124.6	22.73%
179	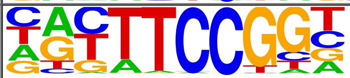	Elk4(ETS)/Hela-Elk4-ChIP-Seq(GSE31477)/Homer	1e-14	-3.414e+01	0.0000	1645.0	19.22%	13465.2	16.00%
180	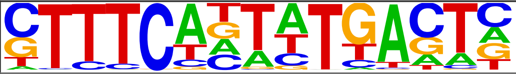	IRF:BATF(IRF:bZIP)/pDC-Irf8-ChIP-Seq(GSE66899)/Homer	1e-14	-3.380e+01	0.0000	433.0	5.06%	2867.1	3.41%
181	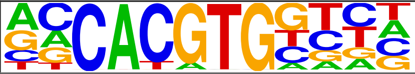	Max(bHLH)/K562-Max-ChIP-Seq(GSE31477)/Homer	1e-14	-3.336e+01	0.0000	1775.0	20.73%	14688.4	17.46%
182	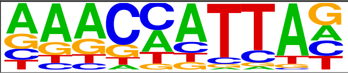	Barx1(Homeobox)/Stomach-Barx1.3xFlag-ChIP-Seq(GSE69483)/Homer	1e-14	-3.310e+01	0.0000	1216.0	14.20%	9627.5	11.44%
183	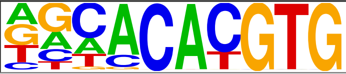	MNT(bHLH)/HepG2-MNT-ChIP-Seq(Encode)/Homer	1e-14	-3.306e+01	0.0000	2447.0	28.58%	20948.8	24.90%

184	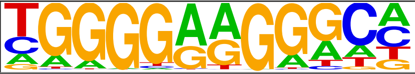	ZNF467(Zf)/HEK293-ZNF467.GFP-ChIP-Seq(GSE58341)/Homer	1e-13	-3.216e+01	0.0000	2221.0	25.94%	18875.6	22.43%
185	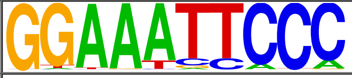	NFkB-p65-Rel(RHD)/ThioMac-LPS-Expression(GSE23622)/Homer	1e-13	-3.208e+01	0.0000	278.0	3.25%	1673.6	1.99%
186	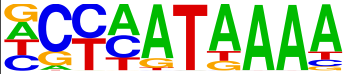	Hoxd13(Homeobox)/ChickenMSG-Hoxd13.Flag-ChIP-Seq(GSE86088)/Homer	1e-13	-3.198e+01	0.0000	3484.0	40.70%	30874.3	36.69%
187	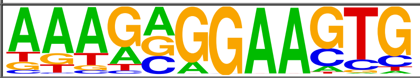	SpiB(ETS)/OCILY3-SPIB-ChIP-Seq(GSE56857)/Homer	1e-13	-3.133e+01	0.0000	756.0	8.83%	5636.3	6.70%
188	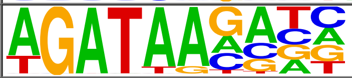	TRPS1(Zf)/MCF7-TRPS1-ChIP-Seq(GSE107013)/Homer	1e-13	-3.108e+01	0.0000	3704.0	43.27%	33049.0	39.28%
189	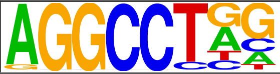	ZFX(Zf)/mES-Zfx-ChIP-Seq(GSE11431)/Homer	1e-13	-3.102e+01	0.0000	2903.0	33.91%	25365.1	30.15%
190	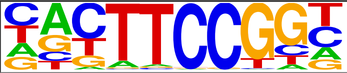	Elk1(ETS)/Hela-Elk1-ChIP-Seq(GSE31477)/Homer	1e-13	-3.072e+01	0.0000	1645.0	19.22%	13608.6	16.17%
191	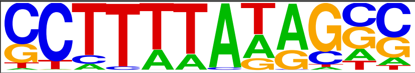	TATA-Box(TBP)/Promoter/Homer	1e-13	-3.058e+01	0.0000	3015.0	35.22%	26456.7	31.44%
192	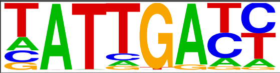	Hnf6b(Homeobox)/LNCaP-Hnf6b-ChIP-Seq(GSE106305)/Homer	1e-13	-3.007e+01	0.0000	1672.0	19.53%	13884.5	16.50%
193	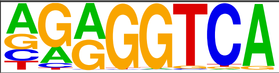	COUP-TFII(NR)/Artia-Nr2f2-ChIP-Seq(GSE46497)/Homer	1e-12	-2.977e+01	0.0000	3825.0	44.68%	34300.0	40.77%
194	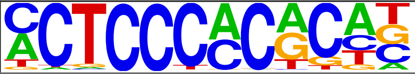	WT1(Zf)/Kidney-WT1-ChIP-Seq(GSE90016)/Homer	1e-12	-2.928e+01	0.0000	1617.0	18.89%	13414.0	15.94%
195	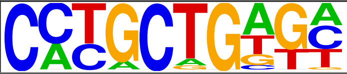	Zic(Zf)/Cerebellum-ZIC1.2-ChIP-Seq(GSE60731)/Homer	1e-12	-2.799e+01	0.0000	2173.0	25.38%	18633.3	22.15%
196	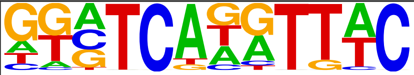	Six1(Homeobox)/Myoblast-Six1-ChIP-Chip(GSE20150)/Homer	1e-12	-2.779e+01	0.0000	868.0	10.14%	6721.3	7.99%
197	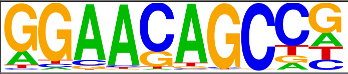	ZNF341(Zf)/EBV-ZNF341-ChIP-Seq(GSE113194)/Homer	1e-11	-2.754e+01	0.0000	1979.0	23.12%	16844.0	20.02%
198	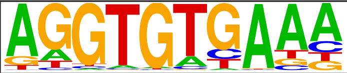	Tbx21(T-box)/GM12878-TBX21-ChIP-Seq(Encode)/Homer	1e-11	-2.690e+01	0.0000	2116.0	24.72%	18156.2	21.58%
199	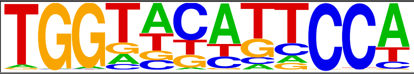	PRDM10(Zf)/HEK293-PRDM10.eGFP-ChIP-Seq(Encode)/Homer	1e-11	-2.679e+01	0.0000	1679.0	19.61%	14098.9	16.76%
200	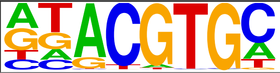	HIF-1b(HLH)/T47D-HIF1b-ChIP-Seq(GSE59937)/Homer	1e-11	-2.677e+01	0.0000	2188.0	25.56%	18837.6	22.39%
201	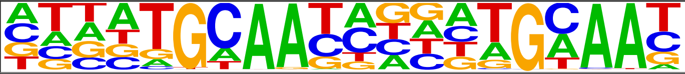	CEBP:CEBP(bZIP)/MEF-Chop-ChIP-Seq(GSE35681)/Homer	1e-11	-2.666e+01	0.0000	432.0	5.05%	3008.9	3.58%
202	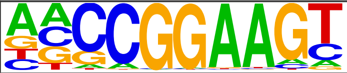	ELF1(ETS)/Jurkat-ELF1-ChIP-Seq(SRA014231)/Homer	1e-11	-2.619e+01	0.0000	1525.0	17.81%	12708.0	15.10%

203	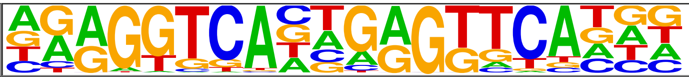	VDR(NR),DR3/GM10855-VDR+vitD-ChIP-Seq(GSE22484)/Homer	1e-11	-2.616e+01	0.0000	559.0	6.53%	4089.8	4.86%
204	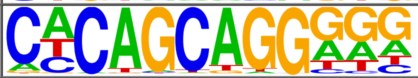	Unknown-ESC-element(?)/mES-Nanog-ChIP-Seq(GSE11724)/Homer	1e-11	-2.591e+01	0.0000	1679.0	19.61%	14140.4	16.81%
205	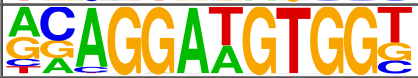	ETS:RUNX(ETS,Runt)/Jurkat-RUNX1-ChIP-Seq(GSE17954)/Homer	1e-11	-2.584e+01	0.0000	381.0	4.45%	2605.6	3.10%
206	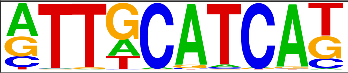	Chop(bZIP)/MEF-Chop-ChIP-Seq(GSE35681)/Homer	1e-10	-2.514e+01	0.0000	656.0	7.66%	4952.0	5.89%
207	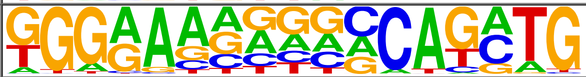	RBPJ:Ebox(?,bHLH)/Panc1-Rbpj1-ChIP-Seq(GSE47459)/Homer	1e-10	-2.500e+01	0.0000	930.0	10.86%	7367.2	8.76%
208	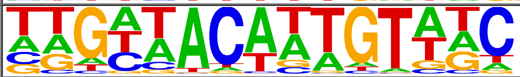	DMRT1(DM)/Testis-DMRT1-ChIP-Seq(GSE64892)/Homer	1e-10	-2.446e+01	0.0000	667.0	7.79%	5068.7	6.02%
209	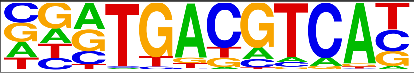	Atf7(bZIP)/3T3L1-Atf7-ChIP-Seq(GSE56872)/Homer	1e-10	-2.428e+01	0.0000	1272.0	14.86%	10479.9	12.46%
210	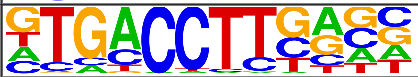	ERRg(NR)/Kidney-ESRRG-ChIP-Seq(GSE104905)/Homer	1e-10	-2.386e+01	0.0000	2048.0	23.92%	17681.2	21.01%
211	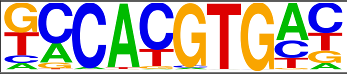	NPAS2(bHLH)/Liver-NPAS2-ChIP-Seq(GSE39860)/Homer	1e-10	-2.382e+01	0.0000	2305.0	26.92%	20101.3	23.89%
212	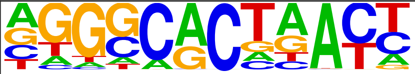	ZNF264(Zf)/HEK293-ZNF264.GFP-ChIP-Seq(GSE58341)/Homer	1e-10	-2.350e+01	0.0000	1668.0	19.48%	14157.4	16.83%
213	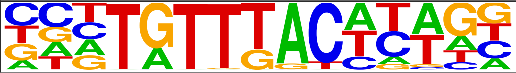	Foxa3(Forkhead)/Liver-Foxa3-ChIP-Seq(GSE77670)/Homer	1e-10	-2.318e+01	0.0000	1041.0	12.16%	8432.7	10.02%
214	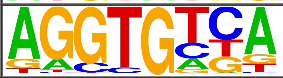	Tbx5(T-box)/HL1-Tbx5.biotin-ChIP-Seq(GSE21529)/Homer	1e-9	-2.299e+01	0.0000	5406.0	63.15%	50305.5	59.79%
215	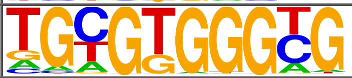	Egr1(Zf)/K562-Egr1-ChIP-Seq(GSE32465)/Homer	1e-9	-2.273e+01	0.0000	1685.0	19.68%	14354.7	17.06%
216	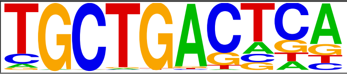	MafA(bZIP)/Islet-MafA-ChIP-Seq(GSE30298)/Homer	1e-9	-2.270e+01	0.0000	2414.0	28.20%	21198.4	25.19%
217	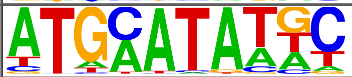	Pit1(Homeobox)/GCrat-Pit1-ChIP-Seq(GSE58009)/Homer	1e-9	-2.262e+01	0.0000	1952.0	22.80%	16850.3	20.03%
218	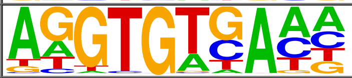	Tbet(T-box)/CD8-Tbet-ChIP-Seq(GSE33802)/Homer	1e-9	-2.210e+01	0.0000	2269.0	26.50%	19862.8	23.61%
219	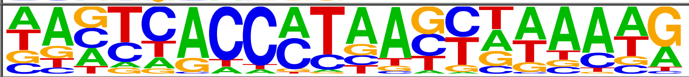	PSE(SNAPc)/K562-mStart-Seq/Homer	1e-9	-2.166e+01	0.0000	1334.0	15.58%	11166.1	13.27%
220	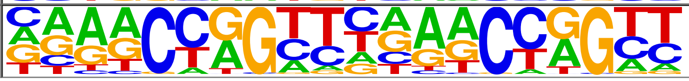	Tcfcp2l1(CP2)/mES-Tcfcp2l1-ChIP-Seq(GSE11431)/Homer	1e-9	-2.140e+01	0.0000	529.0	6.18%	3965.3	4.71%

221	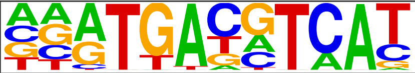	CREB5(bZIP)/LNCaP-CREB5.V5-ChIP-Seq(GSE137775)/Homer	1e-9	-2.140e+01	0.0000	1045.0	12.21%	8542.4	10.15%	
222	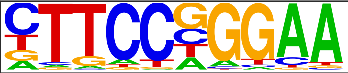	Stat3(Stat)/mES-Stat3-ChIP-Seq(GSE11431)/Homer	1e-9	-2.128e+01	0.0000	1530.0	17.87%	12992.3	15.44%	
223	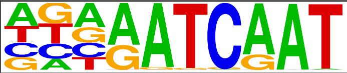	Cux2(Homeobox)/Liver-Cux2-ChIP-Seq(GSE35985)/Homer	1e-9	-2.079e+01	0.0000	792.0	9.25%	6294.9	7.48%	
224	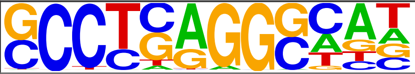	AP-2gamma(AP2)/MCF7-TFAP2C-ChIP-Seq(GSE21234)/Homer	1e-8	-2.030e+01	0.0000	2475.0	28.91%	21927.1	26.06%	
225	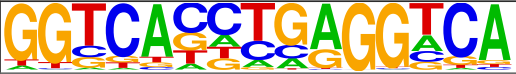	THRb(NR)/HepG2-THRb.Flag-ChIP-Seq(Encode)/Homer	1e-8	-2.006e+01	0.0000	1596.0	18.64%	13669.4	16.25%	
226	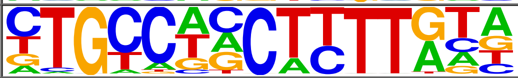	ZNF7(Zf)/HepG2-ZNF7.Flag-ChIP-Seq(Encode)/Homer	1e-8	-2.001e+01	0.0000	1416.0	16.54%	12003.3	14.27%	
227	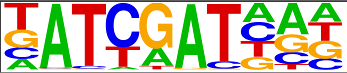	CUX1(Homeobox)/K562-CUX1-ChIP-Seq(GSE92882)/Homer	1e-8	-2.001e+01	0.0000	1127.0	13.16%	9349.2	11.11%	
228	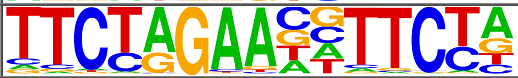	HRE(HSF)/Striatum-HSF1-ChIP-Seq(GSE38000)/Homer	1e-8	-1.995e+01	0.0000	705.0	8.24%	5552.4	6.60%	
229	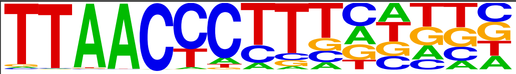	ZNF652/HepG2-ZNF652.Flag-ChIP-Seq(Encode)/Homer	1e-8	-1.986e+01	0.0000	623.0	7.28%	4832.5	5.74%	
230	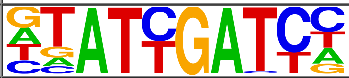	HNF6(Homeobox)/Liver-Hnf6-ChIP-Seq(ERP000394)/Homer	1e-8	-1.893e+01	0.0000	985.0	11.51%	8106.9	9.64%	
231	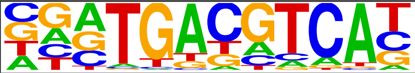	Atf2(bZIP)/3T3L1-Atf2-ChIP-Seq(GSE56872)/Homer	1e-8	-1.868e+01	0.0000	939.0	10.97%	7700.4	9.15%	
232	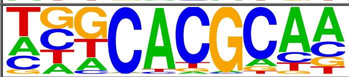	Arnt:Ahr(bHLH)/MCF7-Arnt-ChIP-Seq(Lo_et_al.)/Homer	1e-8	-1.861e+01	0.0000	1433.0	16.74%	12234.9	14.54%	
233	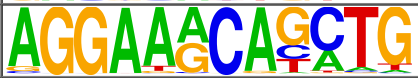	ETS:E-box(ETS,bHLH)/HPC7-Scl-ChIP-Seq(GSE22178)/Homer	1e-7	-1.778e+01	0.0000	299.0	3.49%	2108.1	2.51%	
234	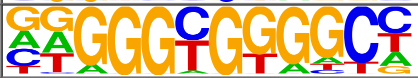	KLF1(Zf)/HUDEP2-KLF1-CutnRun(GSE136251)/Homer	1e-7	-1.773e+01	0.0000	2399.0	28.02%	21376.8	25.41%	
235	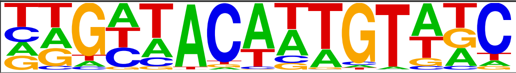	DMRT6(DM)/Testis-DMRT6-ChIP-Seq(GSE60440)/Homer	1e-7	-1.759e+01	0.0000	556.0	6.49%	4321.9	5.14%	
236	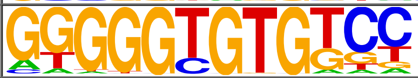	KLF10(Zf)/HEK293-KLF10.GFP-ChIP-Seq(GSE58341)/Homer	1e-7	-1.730e+01	0.0000	1897.0	22.16%	16650.2	19.79%	
237	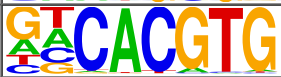	CLOCK(bHLH)/Liver-Clock-ChIP-Seq(GSE39860)/Homer	1e-7	-1.704e+01	0.0000	1285.0	15.01%	10949.1	13.01%	
238	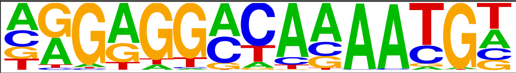	ZNF675(Zf)/HEK293-ZNF675.GFP-ChIP-Seq(GSE58341)/Homer	1e-7	-1.695e+01	0.0000	403.0	4.71%	3011.1	3.58%	
239	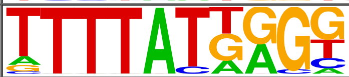	HOXB13(Homeobox)/ProstateTumor-HOXB13-ChIP-Seq(GSE56288)/Homer	1e-7	-1.691e+01	0.0000	2443.0	28.54%	21853.2	25.97%	

240	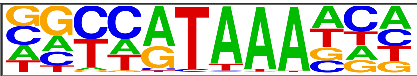	CDX4(Homeobox)/ZebrafishEmbryos-Cdx4.Myc-ChIP-Seq(GSE48254)/Homer	1e-7	-1.689e+01	0.0000	2121.0	24.78%	18793.3	22.34%
241	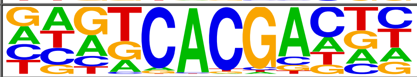	Npas4(bHLH)/Neuron-Npas4-ChIP-Seq(GSE127793)/Homer	1e-7	-1.658e+01	0.0000	1679.0	19.61%	14646.9	17.41%
242	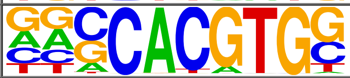	c-Myc(bHLH)/mES-cMyc-ChIP-Seq(GSE11431)/Homer	1e-7	-1.645e+01	0.0000	1270.0	14.83%	10842.8	12.89%
243	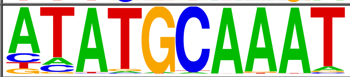	Oct2(POU,Homeobox)/Bcell-Oct2-ChIP-Seq(GSE21512)/Homer	1e-7	-1.634e+01	0.0000	587.0	6.86%	4639.1	5.51%
244	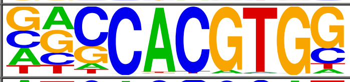	n-Myc(bHLH)/mES-nMyc-ChIP-Seq(GSE11431)/Homer	1e-7	-1.626e+01	0.0000	1671.0	19.52%	14591.0	17.34%
245	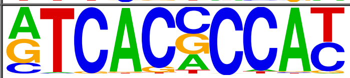	Srebp1a(bHLH)/HepG2-Srebp1a-ChIP-Seq(GSE31477)/Homer	1e-7	-1.618e+01	0.0000	560.0	6.54%	4406.0	5.24%
246	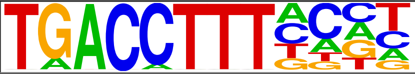	Nur77(NR)/K562-NR4A1-ChIP-Seq(GSE31363)/Homer	1e-7	-1.617e+01	0.0000	514.0	6.00%	4001.2	4.76%
247	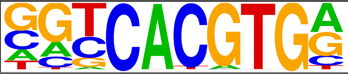	USF1(bHLH)/GM12878-Usf1-ChIP-Seq(GSE32465)/Homer	1e-6	-1.609e+01	0.0000	1131.0	13.21%	9578.5	11.38%
248	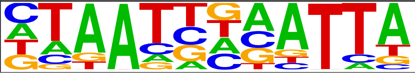	Prop1(Homeobox)/GHFT1-PROP1.biotin-ChIP-Seq(GSE77302)/Homer	1e-6	-1.587e+01	0.0000	1177.0	13.75%	10014.3	11.90%
249	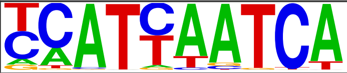	Pdx1(Homeobox)/Islet-Pdx1-ChIP-Seq(SRA008281)/Homer	1e-6	-1.573e+01	0.0000	2056.0	24.02%	18257.0	21.70%
250	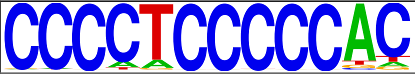	Zfp281(Zf)/ES-Zfp281-ChIP-Seq(GSE81042)/Homer	1e-6	-1.541e+01	0.0000	762.0	8.90%	6243.1	7.42%
251	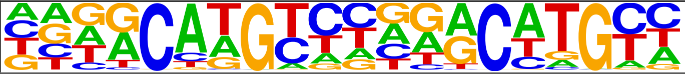	p63(p53)/Keratinocyte-p63-ChIP-Seq(GSE17611)/Homer	1e-6	-1.533e+01	0.0000	938.0	10.96%	7846.3	9.33%
252	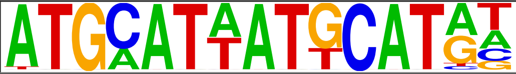	OCT:OCT-short(POU,Homeobox)/NPC-OCT6-ChIP-Seq(GSE43916)/Homer	1e-6	-1.512e+01	0.0000	1423.0	16.62%	12341.8	14.67%
253	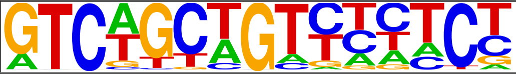	ZNF317(Zf)/HEK293-ZNF317.GFP-ChIP-Seq(GSE58341)/Homer	1e-6	-1.506e+01	0.0000	276.0	3.22%	1982.4	2.36%
254	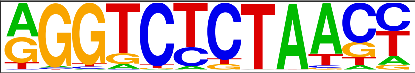	PRDM14(Zf)/H1-PRDM14-ChIP-Seq(GSE22767)/Homer	1e-6	-1.499e+01	0.0000	814.0	9.51%	6733.5	8.00%
255	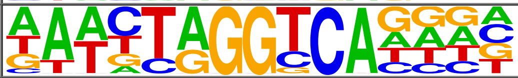	RORa(NR)/Liver-Rora-ChIP-Seq(GSE101115)/Homer	1e-6	-1.495e+01	0.0000	415.0	4.85%	3176.1	3.77%
256	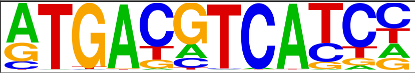	c-Jun-CRE(bZIP)/K562-cJun-ChIP-Seq(GSE31477)/Homer	1e-6	-1.463e+01	0.0000	847.0	9.89%	7051.0	8.38%
257	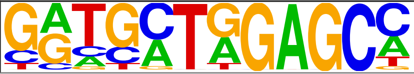	ZNF415(Zf)/HEK293-ZNF415.GFP-ChIP-Seq(GSE58341)/Homer	1e-6	-1.445e+01	0.0000	1337.0	15.62%	11580.3	13.76%

258	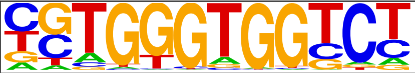	Gli2(Zf)/GM2-Gli2-ChIP-Chip(GSE112702)/Homer	1e-6	-1.441e+01	0.0000	681.0	7.95%	5556.2	6.60%
259	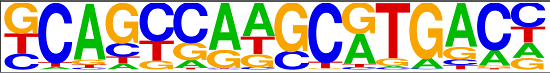	PAX5(Paired,Homeobox)/GM12878-PAX5-ChIP-Seq(GSE32465)/Homer	1e-6	-1.419e+01	0.0000	978.0	11.42%	8271.2	9.83%
260	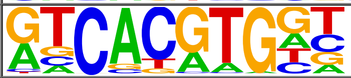	Usf2(bHLH)/C2C12-Usf2-ChIP-Seq(GSE36030)/Homer	1e-6	-1.416e+01	0.0000	864.0	10.09%	7228.4	8.59%
261	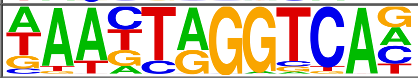	RORg(NR)/Liver-Rorc-ChIP-Seq(GSE101115)/Homer	1e-6	-1.416e+01	0.0000	269.0	3.14%	1946.1	2.31%
262	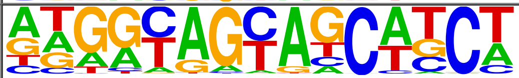	PRDM9(Zf)/Testis-DMC1-ChIP-Seq(GSE35498)/Homer	1e-6	-1.410e+01	0.0000	1061.0	12.39%	9040.3	10.74%
263	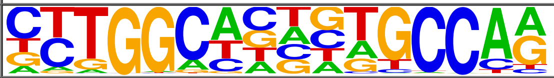	NF1(CTF)/LNCAP-NF1-ChIP-Seq(Unpublished)/Homer	1e-6	-1.410e+01	0.0000	1424.0	16.63%	12414.1	14.75%
264	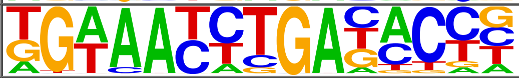	Six4(Homeobox)/MCF7-SIX4-ChIP-Seq(Encode)/Homer	1e-6	-1.407e+01	0.0000	241.0	2.82%	1713.9	2.04%
265	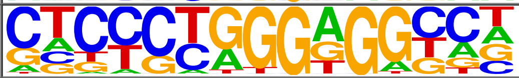	GLIS3(Zf)/Thyroid-Glis3.GFP-ChIP-Seq(GSE103297)/Homer	1e-6	-1.383e+01	0.0000	3121.0	36.46%	28608.2	34.00%
266	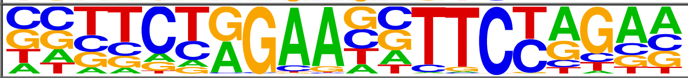	HRE(HSF)/HepG2-HSF1-ChIP-Seq(GSE31477)/Homer	1e-5	-1.379e+01	0.0000	494.0	5.77%	3911.0	4.65%
267	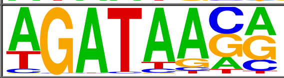	GATA3(Zf)/iTreg-Gata3-ChIP-Seq(GSE20898)/Homer	1e-5	-1.374e+01	0.0000	2772.0	32.38%	25250.3	30.01%
268	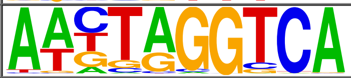	RORgt(NR)/EL4-RORgt.Flag-ChIP-Seq(GSE56019)/Homer	1e-5	-1.366e+01	0.0000	371.0	4.33%	2835.0	3.37%
269	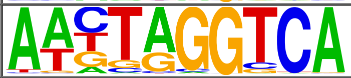	RORgt(NR)/EL4-RORgt.Flag-ChIP-Seq(GSE56019)/Homer	1e-5	-1.366e+01	0.0000	371.0	4.33%	2835.0	3.37%
270	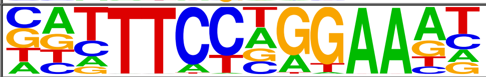	STAT1(Stat)/HelaS3-STAT1-ChIP-Seq(GSE12782)/Homer	1e-5	-1.338e+01	0.0000	958.0	11.19%	8130.1	9.66%
271	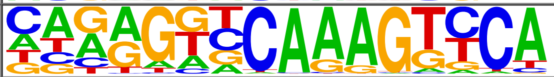	HNF4a(NR),DR1/HepG2-HNF4a-ChIP-Seq(GSE25021)/Homer	1e-5	-1.330e+01	0.0000	1298.0	15.16%	11287.2	13.42%
272	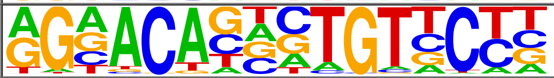	ARE(NR)/LNCAP-AR-ChIP-Seq(GSE27824)/Homer	1e-5	-1.280e+01	0.0000	797.0	9.31%	6684.6	7.94%
273	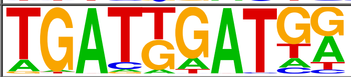	HOXA1(Homeobox)/mES-Hoxa1-ChIP-Seq(SRP084292)/Homer	1e-5	-1.275e+01	0.0000	578.0	6.75%	4701.6	5.59%
274	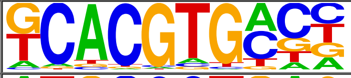	bHLHE41(bHLH)/proB-Bhlhe41-ChIP-Seq(GSE93764)/Homer	1e-5	-1.252e+01	0.0000	2090.0	24.41%	18819.2	22.37%
275	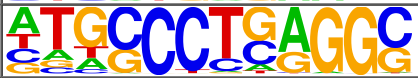	AP-2alpha(AP2)/Hela-AP2alpha-ChIP-Seq(GSE31477)/Homer	1e-5	-1.246e+01	0.0000	1904.0	22.24%	17055.3	20.27%
276	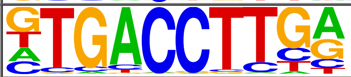	Esrrb(NR)/mES-Esrrb-ChIP-Seq(GSE11431)/Homer	1e-5	-1.245e+01	0.0000	1608.0	18.78%	14254.8	16.94%

277	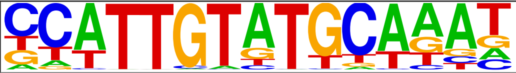	Oct4:Sox17(POU,Homeobox,HMG)/F9-Sox17-ChIP-Seq(GSE44553)/Homer	1e-5	-1.227e+01	0.0000	337.0	3.94%	2584.3	3.07%
278	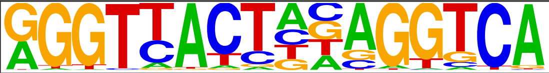	LXRE(NR),DR4/RAW-LXRb.biotin-ChIP-Seq(GSE21512)/Homer	1e-5	-1.224e+01	0.0000	165.0	1.93%	1126.4	1.34%
279	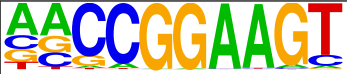	ETS(ETS)/Promoter/Homer	1e-5	-1.206e+01	0.0000	1019.0	11.90%	8768.6	10.42%
280	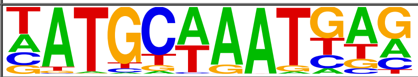	Oct6(POU,Homeobox)/NPC-Pou3f1-ChIP-Seq(GSE35496)/Homer	1e-5	-1.198e+01	0.0000	825.0	9.64%	6983.0	8.30%
281	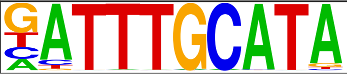	Oct11(POU,Homeobox)/NCIH1048-POU2F3-ChIP-seq(GSE115123)/Homer	1e-5	-1.179e+01	0.0000	655.0	7.65%	5440.7	6.47%
282	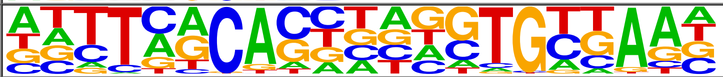	Brachyury(T-box)/Mesoendoderm-Brachyury-ChIP-exo(GSE54963)/Homer	1e-5	-1.175e+01	0.0000	726.0	8.48%	6089.5	7.24%
283	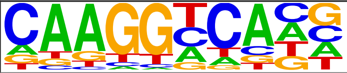	SF1(NR)/H295R-Nr5a1-ChIP-Seq(GSE44220)/Homer	1e-5	-1.157e+01	0.0000	1276.0	14.90%	11194.7	13.31%
284	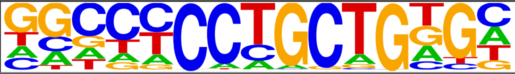	Zic3(Zf)/mES-Zic3-ChIP-Seq(GSE37889)/Homer	1e-4	-1.150e+01	0.0000	1530.0	17.87%	13587.2	16.15%
285	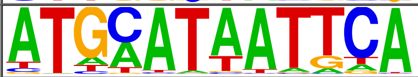	Pit1+1bp(Homeobox)/GCrat-Pit1-ChIP-Seq(GSE58009)/Homer	1e-4	-1.147e+01	0.0000	670.0	7.83%	5592.3	6.65%
286	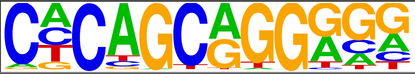	Zic2(Zf)/ESC-Zic2-ChIP-Seq(SRP197560)/Homer	1e-4	-1.132e+01	0.0000	1204.0	14.06%	10537.9	12.52%
287	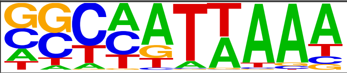	Unknown(Homeobox)/Limb-p300-ChIP-Seq/Homer	1e-4	-1.107e+01	0.0000	1529.0	17.86%	13610.3	16.18%
288	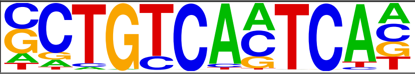	Pbx3(Homeobox)/GM12878-PBX3-ChIP-Seq(GSE32465)/Homer	1e-4	-1.093e+01	0.0000	652.0	7.62%	5455.6	6.48%
289	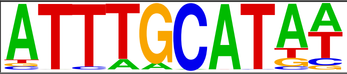	Oct4(POU,Homeobox)/mES-Oct4-ChIP-Seq(GSE11431)/Homer	1e-4	-1.080e+01	0.0000	914.0	10.68%	7871.2	9.36%
290	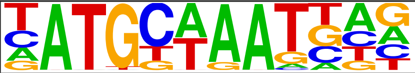	Brn1(POU,Homeobox)/NPC-Brn1-ChIP-Seq(GSE35496)/Homer	1e-4	-1.075e+01	0.0000	612.0	7.15%	5100.1	6.06%
291	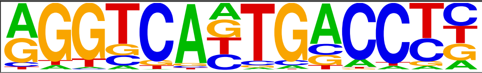	FXR(NR),IR1/Liver-FXR-ChIP-Seq(Chong_et_al.)/Homer	1e-4	-1.075e+01	0.0000	969.0	11.32%	8384.6	9.97%
292	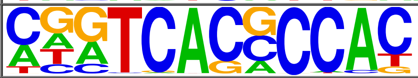	Srebp2(bHLH)/HepG2-Srebp2-ChIP-Seq(GSE31477)/Homer	1e-4	-1.066e+01	0.0000	363.0	4.24%	2868.5	3.41%
293	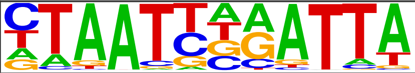	Phox2a(Homeobox)/Neuron-Phox2a-ChIP-Seq(GSE31456)/Homer	1e-4	-1.043e+01	0.0000	715.0	8.35%	6057.8	7.20%
294	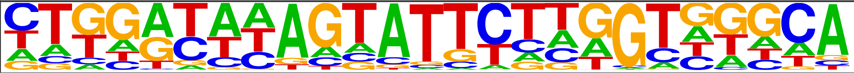	ZNF136(Zf)/HEK293-ZNF136.GFP-ChIP-Seq(GSE58341)/Homer	1e-4	-1.039e+01	0.0000	243.0	2.84%	1829.2	2.17%

295	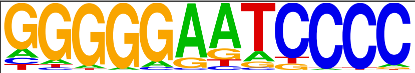	NFkB-p50,p52(RHD)/Monocyte-p50-ChIP-Chip(Schreiber_et_al.)/Homer	1e-4	-1.028e+01	0.0001	287.0	3.35%	2214.9	2.63%
296	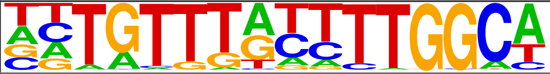	NF1:FOXA1(CTF,Forkhead)/LNCAP-FOXA1-ChIP-Seq(GSE27824)/Homer	1e-4	-1.027e+01	0.0001	375.0	4.38%	2989.2	3.55%
297	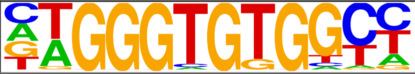	EKLF(Zf)/Erythrocyte-Klf1-ChIP-Seq(GSE20478)/Homer	1e-4	-1.022e+01	0.0001	799.0	9.33%	6842.1	8.13%
298	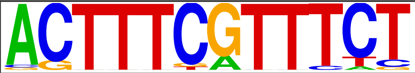	T1ISRE(IRF)/ThioMac-Ifnb-Expression/Homer	1e-4	-1.006e+01	0.0001	57.0	0.67%	315.4	0.37%
299	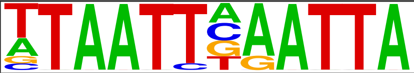	Phox2b(Homeobox)/CLBGA-PHOX2B-ChIP-Seq(GSE90683)/Homer	1e-4	-1.005e+01	0.0001	378.0	4.42%	3024.7	3.59%
300	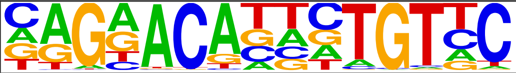	GRE(NR),IR3/RAW264.7-GRE-ChIP-Seq(Unpublished)/Homer	1e-4	-1.004e+01	0.0001	671.0	7.84%	5675.3	6.75%
301	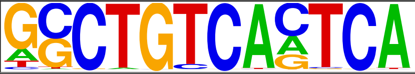	PBX1(Homeobox)/MCF7-PBX1-ChIP-Seq(GSE28007)/Homer	1e-4	-9.938e+00	0.0001	248.0	2.90%	1886.2	2.24%
302	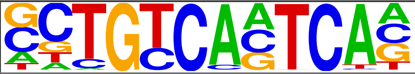	Pknox1(Homeobox)/ES-Prep1-ChIP-Seq(GSE63282)/Homer	1e-4	-9.768e+00	0.0001	609.0	7.11%	5122.6	6.09%
303	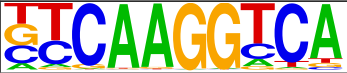	Nr5a2(NR)/Pancreas-LRH1-ChIP-Seq(GSE34295)/Homer	1e-4	-9.664e+00	0.0001	1806.0	21.10%	16352.4	19.44%
304	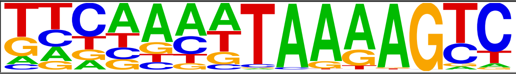	MYNN(Zf)/HEK293-MYNN.eGFP-ChIP-Seq(Encode)/Homer	1e-4	-9.620e+00	0.0001	644.0	7.52%	5450.6	6.48%
305	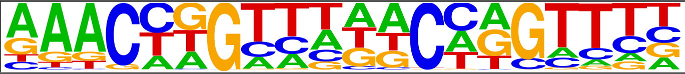	GRHL2(CP2)/HBE-GRHL2-ChIP-Seq(GSE46194)/Homer	1e-4	-9.377e+00	0.0001	895.0	10.45%	7783.2	9.25%
306	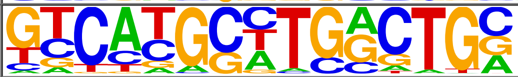	Pax8(Paired,Homeobox)/Thyroid-Pax8-ChIP-Seq(GSE26938)/Homer	1e-3	-9.201e+00	0.0001	867.0	10.13%	7534.9	8.96%
307	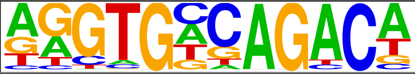	Tbox:Smad(T-box,MAD)/ESCd5-Smad2_3-ChIP-Seq(GSE29422)/Homer	1e-3	-8.922e+00	0.0002	514.0	6.00%	4299.3	5.11%
308	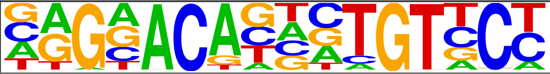	GRE(NR),IR3/A549-GR-ChIP-Seq(GSE32465)/Homer	1e-3	-8.575e+00	0.0003	395.0	4.61%	3238.6	3.85%
309	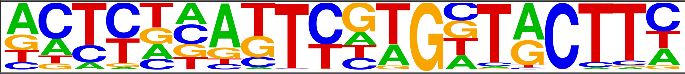	Mouse_Recombination_Hotspot(Zf)/Testis-DMC1-ChIP-Seq(GSE24438)/Homer	1e-3	-8.531e+00	0.0003	179.0	2.09%	1334.4	1.59%
310	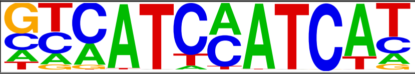	HOXA2(Homeobox)/mES-Hoxa2-ChIP-Seq(Donaldson_et_al.)/Homer	1e-3	-8.469e+00	0.0003	241.0	2.82%	1873.1	2.23%
311	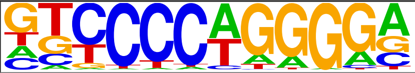	EBF1(EBF)/Near-E2A-ChIP-Seq(GSE21512)/Homer	1e-3	-8.219e+00	0.0004	2709.0	31.64%	25171.5	29.92%
312	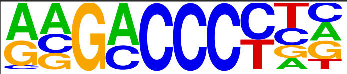	LRF(Zf)/Erythroblasts-ZBTB7A-ChIP-Seq(GSE74977)/Homer	1e-3	-7.845e+00	0.0006	2425.0	28.33%	22469.2	26.71%

313	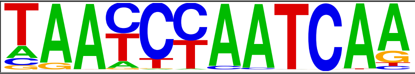	Duxbl(Homeobox)/NIH3T3-Duxbl.HA-ChIP-Seq(GSE119782)/Homer	1e-3	-7.831e+00	0.0006	146.0	1.71%	1072.6	1.27%
314	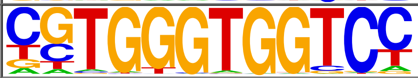	GLI3(Zf)/Limb-GLI3-ChIP-Chip(GSE11077)/Homer	1e-3	-7.653e+00	0.0007	310.0	3.62%	2515.1	2.99%
315	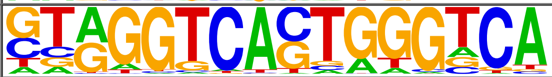	Reverb(NR),DR2/RAW-Reverba.biotin-ChIP-Seq(GSE45914)/Homer	1e-3	-7.476e+00	0.0008	392.0	4.58%	3262.3	3.88%
316	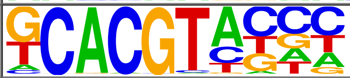	HIF2a(bHLH)/785_O-HIF2a-ChIP-Seq(GSE34871)/Homer	1e-3	-7.452e+00	0.0008	734.0	8.57%	6412.3	7.62%
317	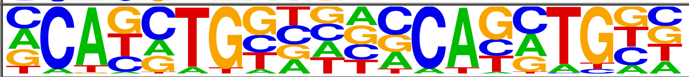	Twist(bHLH)/HMLE-TWIST1-ChIP-Seq(Chang_et_al)/Homer	1e-3	-7.412e+00	0.0008	346.0	4.04%	2849.2	3.39%
318	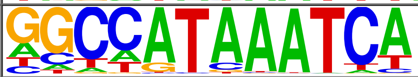	Hoxc9(Homeobox)/Ainv15-Hoxc9-ChIP-Seq(GSE21812)/Homer	1e-3	-7.359e+00	0.0009	1214.0	14.18%	10930.6	12.99%
319	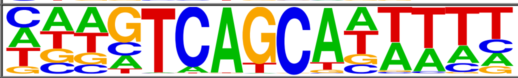	MafF(bZIP)/HepG2-MafF-ChIP-Seq(GSE31477)/Homer	1e-3	-7.308e+00	0.0009	689.0	8.05%	6003.5	7.14%
320	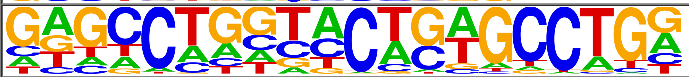	ZNF322(Zf)/HEK293-ZNF322.GFP-ChIP-Seq(GSE58341)/Homer	1e-3	-7.239e+00	0.0010	781.0	9.12%	6865.8	8.16%
321	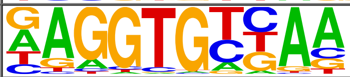	Tbx6(T-box)/ESC-Tbx6-ChIP-Seq(GSE93524)/Homer	1e-3	-7.188e+00	0.0010	2044.0	23.88%	18872.4	22.43%
322	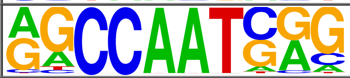	NFY(CCAAT)/Promoter/Homer	1e-3	-6.986e+00	0.0013	1840.0	21.49%	16935.9	20.13%
323	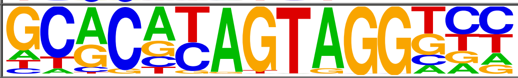	ZKSCAN1(Zf)/HepG2-ZKSCAN1-ChIP-Seq(Encode)/Homer	1e-3	-6.975e+00	0.0013	82.0	0.96%	558.1	0.66%
324	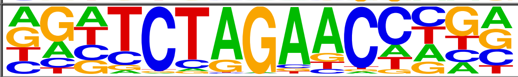	ZBTB12(Zf)/HEK293-ZBTB12.GFP-ChIP-Seq(GSE58341)/Homer	1e-2	-6.865e+00	0.0014	1076.0	12.57%	9667.5	11.49%
325	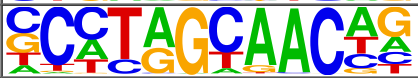	Rfx5(HTH)/GM12878-Rfx5-ChIP-Seq(GSE31477)/Homer	1e-2	-6.703e+00	0.0017	937.0	10.94%	8369.7	9.95%
326	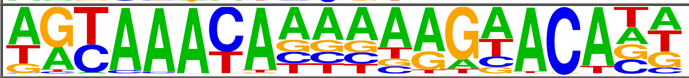	FOXA1:AR(Forkhead,NR)/LNCAP-AR-ChIP-Seq(GSE27824)/Homer	1e-2	-6.663e+00	0.0017	220.0	2.57%	1753.7	2.08%
327	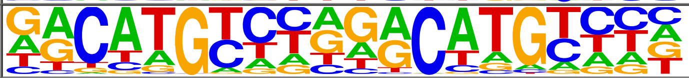	p53(p53)/Saos-p53-ChIP-Seq(GSE15780)/Homer	1e-2	-6.629e+00	0.0018	253.0	2.96%	2048.5	2.43%
328	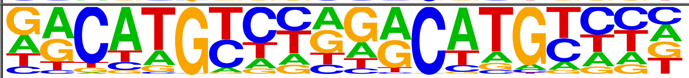	p53(p53)/Saos-p53-ChIP-Seq/Homer	1e-2	-6.629e+00	0.0018	253.0	2.96%	2048.5	2.43%
329	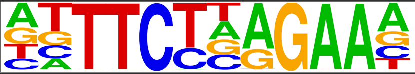	STAT5(Stat)/mCD4+-Stat5-ChIP-Seq(GSE12346)/Homer	1e-2	-6.600e+00	0.0018	1061.0	12.39%	9548.5	11.35%
330	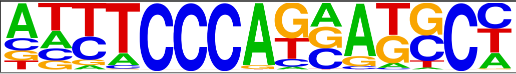	ZNF143|STAF(Zf)/CUTLL-ZNF143-ChIP-Seq(GSE29600)/Homer	1e-2	-6.082e+00	0.0030	1044.0	12.19%	9433.0	11.21%

331	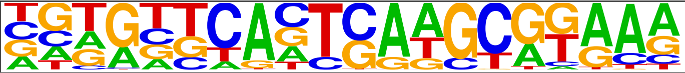	PAX6(Paired,Homeobox)/Forebrain-Pax6-ChIP-Seq(GSE66961)/Homer	1e-2	-6.050e+00	0.0031	221.0	2.58%	1786.7	2.12%	
332	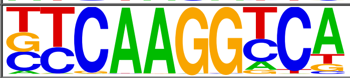	Nr5a2(NR)/mES-Nr5a2-ChIP-Seq(GSE19019)/Homer	1e-2	-5.946e+00	0.0035	1377.0	16.08%	12614.5	14.99%	
333	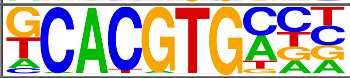	bHLHE40(bHLH)/HepG2-BHLHE40-ChIP-Seq(GSE31477)/Homer	1e-2	-5.673e+00	0.0045	633.0	7.39%	5595.8	6.65%	
334	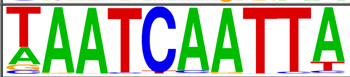	Pax7(Paired,Homeobox)/Myoblast-Pax7-ChIP-Seq(GSE25064)/Homer	1e-2	-5.588e+00	0.0049	157.0	1.83%	1236.5	1.47%	
335	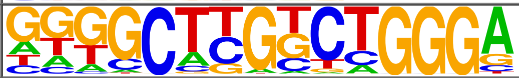	Zfp809(Zf)/ES-Zfp809-ChIP-Seq(GSE70799)/Homer	1e-2	-5.557e+00	0.0051	520.0	6.07%	4549.4	5.41%	
336	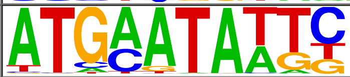	Brn2(POU,Homeobox)/NPC-Brn2-ChIP-Seq(GSE35496)/Homer	1e-2	-5.468e+00	0.0055	189.0	2.21%	1524.6	1.81%	
337	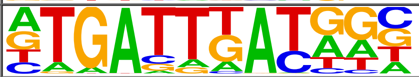	PBX2(Homeobox)/K562-PBX2-ChIP-Seq(Encode)/Homer	1e-2	-5.258e+00	0.0068	1794.0	20.96%	16692.6	19.84%	
338	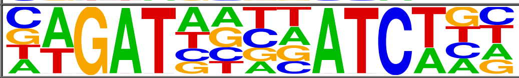	GATA(Zf),IR4/iTreg-Gata3-ChIP-Seq(GSE20898)/Homer	1e-2	-5.247e+00	0.0069	159.0	1.86%	1266.6	1.51%	
339	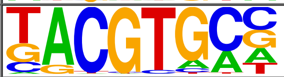	HIF-1a(bHLH)/MCF7-HIF1a-ChIP-Seq(GSE28352)/Homer	1e-2	-5.198e+00	0.0072	492.0	5.75%	4313.1	5.13%	
340	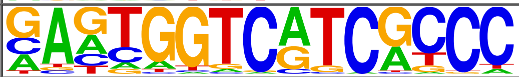	ZNF669(Zf)/HEK293-ZNF669.GFP-ChIP-Seq(GSE58341)/Homer	1e-2	-5.139e+00	0.0076	179.0	2.09%	1448.8	1.72%	
341	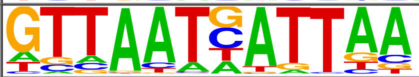	HNF1b(Homeobox)/PDAC-HNF1B-ChIP-Seq(GSE64557)/Homer	1e-2	-5.138e+00	0.0076	256.0	2.99%	2142.8	2.55%	
342	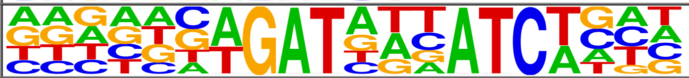	GATA(Zf),IR3/iTreg-Gata3-ChIP-Seq(GSE20898)/Homer	1e-2	-5.114e+00	0.0077	304.0	3.55%	2581.8	3.07%	
343	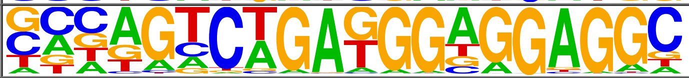	ZSCAN22(Zf)/HEK293-ZSCAN22.GFP-ChIP-Seq(GSE58341)/Homer	1e-2	-5.026e+00	0.0084	215.0	2.51%	1776.5	2.11%	
344	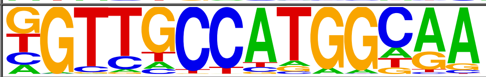	Rfx1(HTH)/NPC-H3K4me1-ChIP-Seq(GSE16256)/Homer	1e-2	-4.721e+00	0.0114	556.0	6.49%	4946.1	5.88%	
345	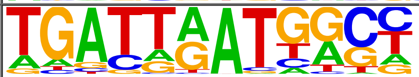	Hoxb4(Homeobox)/ES-Hoxb4-ChIP-Seq(GSE34014)/Homer	1e-2	-4.671e+00	0.0119	470.0	5.49%	4146.0	4.93%	
