# Supplementary material for: Single cell cortical bone transcriptomics define novel osteolineage gene sets altered in chronic kidney disease
Source: Front Endocrinol (Lausanne). 2023 Jan 26;14:1063083. doi: 10.3389/fendo.2023.1063083 (PMC9910177; doi:10.3389/fendo.2023.1063083)
Supplement: Supplementary file 3 [file Table_1.docx]

**Supplementary Table 1. Osteoblast and osteocyte markers.** Table lists the markers that identified Tnc-Mmp13 osteoblasts, osteoblasts, and osteocytes. The pct.1 is the percentage of cells in the cluster where the gene is detected, whereas pct.2 is the percentage of cells where the gene is detected among all the other cells.

| Tnc_Mmp13 Osteoblasts | | | | | Osteoblasts | | | | | Osteocytes | | | | |
| --- | --- | --- | --- | --- | --- | --- | --- | --- | --- | --- | --- | --- | --- | --- |
| markers | log2FC | pct.1 | pct.2 | p_val_adj | markers | log2FC | pct.1 | pct.2 | p_val_adj | markers | log2FC | pct.1 | pct.2 | p_val_adj |
| Mmp13 | 4.32 | 0.669 | 0.092 | 1.77E-36 | Smpd3 | 3.46 | 0.973 | 0.108 | 1.38E-75 | Phex | 3.91 | 1 | 0.19 | 2.87E-34 |
| Tnc | 3.88 | 0.855 | 0.189 | 6.05E-58 | Bglap | 3.36 | 0.973 | 0.384 | 4.14E-45 | Dmp1 | 3.66 | 0.923 | 0.211 | 2.68E-14 |
| Igfbp5 | 3.59 | 0.589 | 0.109 | 1.09E-24 | Bglap2 | 3.33 | 0.955 | 0.286 | 2.47E-45 | Cd109 | 3.02 | 0.962 | 0.144 | 5.61E-36 |
| Serpine2 | 3.43 | 0.96 | 0.305 | 3.32E-59 | Col11a2 | 3.26 | 1 | 0.165 | 1.17E-69 | Ackr3 | 2.86 | 0.923 | 0.069 | 6.51E-26 |
| Spp1 | 3.10 | 0.79 | 0.364 | 5.47E-29 | Col1a1 | 3.15 | 1 | 0.547 | 2.79E-63 | Ptprz1 | 2.73 | 1 | 0.194 | 3.43E-19 |
| Serping1 | 2.98 | 0.548 | 0.102 | 4.46E-27 | Col1a2 | 2.82 | 1 | 0.499 | 3.35E-55 | Car12 | 2.62 | 0.731 | 0.035 | 4.23E-18 |
| Lifr | 2.84 | 0.798 | 0.421 | 9.60E-43 | Col11a1 | 2.76 | 0.991 | 0.243 | 1.14E-56 | Ramp1 | 2.59 | 0.962 | 0.305 | 2.05E-19 |
| Postn | 2.70 | 0.637 | 0.109 | 8.47E-35 | Serpinf1 | 2.68 | 0.973 | 0.307 | 6.81E-52 | Bambi | 2.55 | 0.923 | 0.386 | 1.48E-14 |
| Cdh11 | 2.57 | 0.766 | 0.243 | 3.61E-52 | Cthrc1 | 2.42 | 0.855 | 0.11 | 7.77E-53 | Dkk1 | 2.54 | 0.923 | 0.067 | 1.17E-19 |
| Gdpd2 | 2.57 | 0.435 | 0.009 | 3.41E-31 | Col5a2 | 2.39 | 1 | 0.378 | 1.24E-55 | Pdgfa | 2.50 | 1 | 0.276 | 5.96E-22 |
| Pdgfrb | 2.53 | 0.589 | 0.054 | 3.39E-42 | Sparc | 2.35 | 1 | 0.65 | 1.05E-51 | Pdpn | 2.29 | 0.885 | 0.067 | 7.27E-20 |
| Gas6 | 2.52 | 0.556 | 0.177 | 6.36E-19 | Ibsp | 2.35 | 0.982 | 0.316 | 3.22E-37 | Wasl | 2.18 | 0.962 | 0.273 | 5.59E-15 |
| Olfml2b | 2.51 | 0.669 | 0.137 | 2.73E-37 | Ifitm5 | 2.29 | 0.873 | 0.126 | 5.79E-48 | Col24a1 | 2.09 | 0.923 | 0.125 | 1.51E-17 |
| Col6a1 | 2.41 | 0.677 | 0.156 | 7.52E-28 | Col5a1 | 2.13 | 0.991 | 0.281 | 2.30E-53 | Ptgis | 2.06 | 1 | 0.307 | 5.67E-13 |
| Col12a1 | 2.37 | 0.637 | 0.118 | 2.49E-29 | Cpz | 2.09 | 0.855 | 0.078 | 3.88E-54 | Bmp2 | 2.05 | 0.846 | 0.113 | 8.36E-15 |
| Lum1 | 2.36 | 0.71 | 0.293 | 2.17E-20 | Ccn1 | 2.08 | 0.718 | 0.256 | 6.98E-16 | Ccn4 | 2.04 | 1 | 0.307 | 6.14E-13 |
| Islr | 2.33 | 0.565 | 0.106 | 6.98E-34 | Cgref1 | 1.96 | 0.827 | 0.101 | 1.93E-49 | Gm41724 | 1.94 | 0.692 | 0.052 | 5.37E-15 |
| Col8a1 | 2.30 | 0.387 | 0.033 | 5.23E-20 | Nupr1 | 1.94 | 0.945 | 0.334 | 1.42E-33 | Spns2 | 1.84 | 0.923 | 0.273 | 7.79E-14 |
| Cfh1 | 2.29 | 0.895 | 0.364 | 3.51E-34 | Fkbp11 | 1.93 | 0.873 | 0.128 | 2.22E-50 | Bmp4 | 1.81 | 0.885 | 0.077 | 1.20E-16 |
| Col6a2 | 2.24 | 0.629 | 0.139 | 1.25E-23 | Rrbp1 | 1.92 | 0.991 | 0.627 | 1.01E-52 | Plpp1 | 1.81 | 1 | 0.246 | 1.07E-11 |
| Loxl1 | 2.20 | 0.5 | 0.043 | 3.96E-32 | Serpinh1 | 1.85 | 1 | 0.558 | 5.32E-43 | Irx5 | 1.78 | 0.962 | 0.069 | 1.22E-21 |
| Wif11 | 2.19 | 0.532 | 0.227 | 1.40E-12 | Timp1 | 1.85 | 0.791 | 0.238 | 1.07E-22 | Myo1b | 1.71 | 0.962 | 0.338 | 7.29E-13 |
| Limch1 | 2.19 | 0.468 | 0.116 | 4.32E-19 | Creb3l1 | 1.84 | 0.891 | 0.185 | 1.24E-44 | Adamts14 | 1.70 | 0.846 | 0.058 | 7.65E-20 |
| Rn18s | 2.18 | 1 | 0.998 | 2.82E-30 | Col22a1 | 1.84 | 0.973 | 0.176 | 2.29E-53 | Cspg4 | 1.58 | 0.923 | 0.171 | 2.58E-11 |
| Cp | 2.18 | 0.661 | 0.132 | 2.62E-33 | Car3 | 1.80 | 0.909 | 0.19 | 1.28E-40 | Ccdc194 | 1.57 | 0.885 | 0.104 | 8.03E-15 |
