# Supplementary material for: Single cell cortical bone transcriptomics define novel osteolineage gene sets altered in chronic kidney disease
Source: Front Endocrinol (Lausanne). 2023 Jan 26;14:1063083. doi: 10.3389/fendo.2023.1063083 (PMC9910177; doi:10.3389/fendo.2023.1063083)
Supplement: Supplementary file 4 [file Table_2.docx]

**Supplementary Table 2:** **Jaccard index analysis.** The Jaccard scores were calculated for the four groups of overlapping differentials expressed genes (DEGs) and differential accessible regions (DARs) based on their up- or down-regulated directions: both up, both down, DEG up & DAR down, and DEG down & DAR up.

| ​ | Cutoffs: FDR < 0.05, \|logFC\| >1​ | |
| --- | --- | --- |
| ​ | Overlap p value​ | jaccard​ |
| DAR_Up and DE_Up​ | 3.25e-35​ | 0.0714​ |
| DAR_Down and DE_Down | 0.00111​ | 0.0296​ |
| DAR_Up and DE_Down​ | 1​ | 0.00309​ |
| DAR_Down and DE_Up​ | 1​ | 0.00605​ |
