# Supplementary figures and images for: Single cell cortical bone transcriptomics define novel osteolineage gene sets altered in chronic kidney disease
Source: Front Endocrinol (Lausanne). 2023 Jan 26;14:1063083. doi: 10.3389/fendo.2023.1063083 (PMC9910177; doi:10.3389/fendo.2023.1063083)

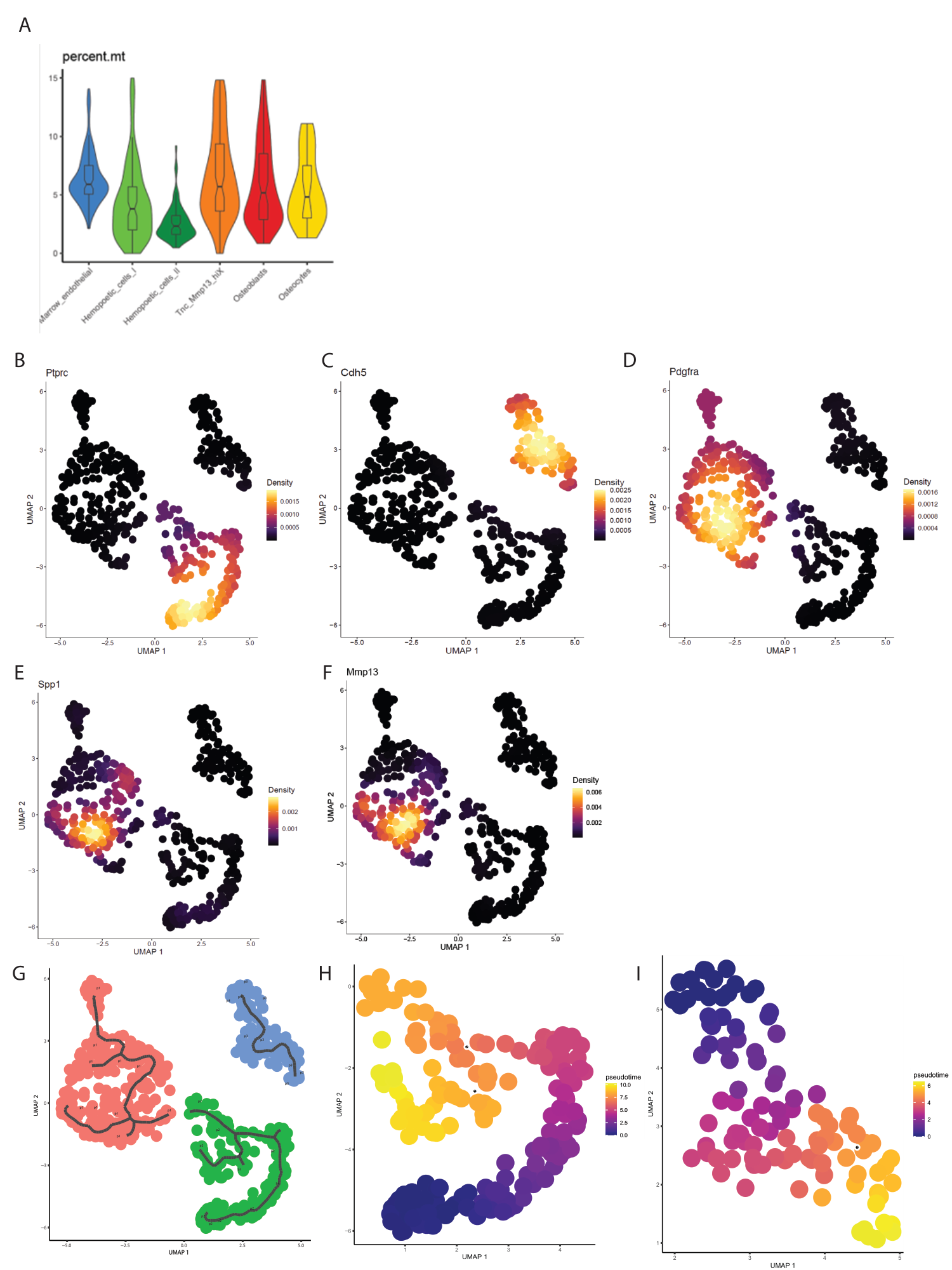

Supplement: Supplementary Figure 1 — (A). Percentage of mitochondrial genes in scRNAseq dataset. (B–F). Expression density plots indicated cells with high transcription of Ptprc (Cd45), Cdh5, Pdgfra, Mmp13, and Spp1. (G). The Monocle algorithm divided the cells based upon the original UMAP into three partitions, as indicated by each color. The partition ‘p1’ identified the osteolineage cells, ‘p2’ corresponded to hematopoietic cells and ‘p3’ represented endothelial cells. (H–I). Examples of trajectory analysis performed on hematopoietic and marrow endothelial cells. [file Image_1.tiff]

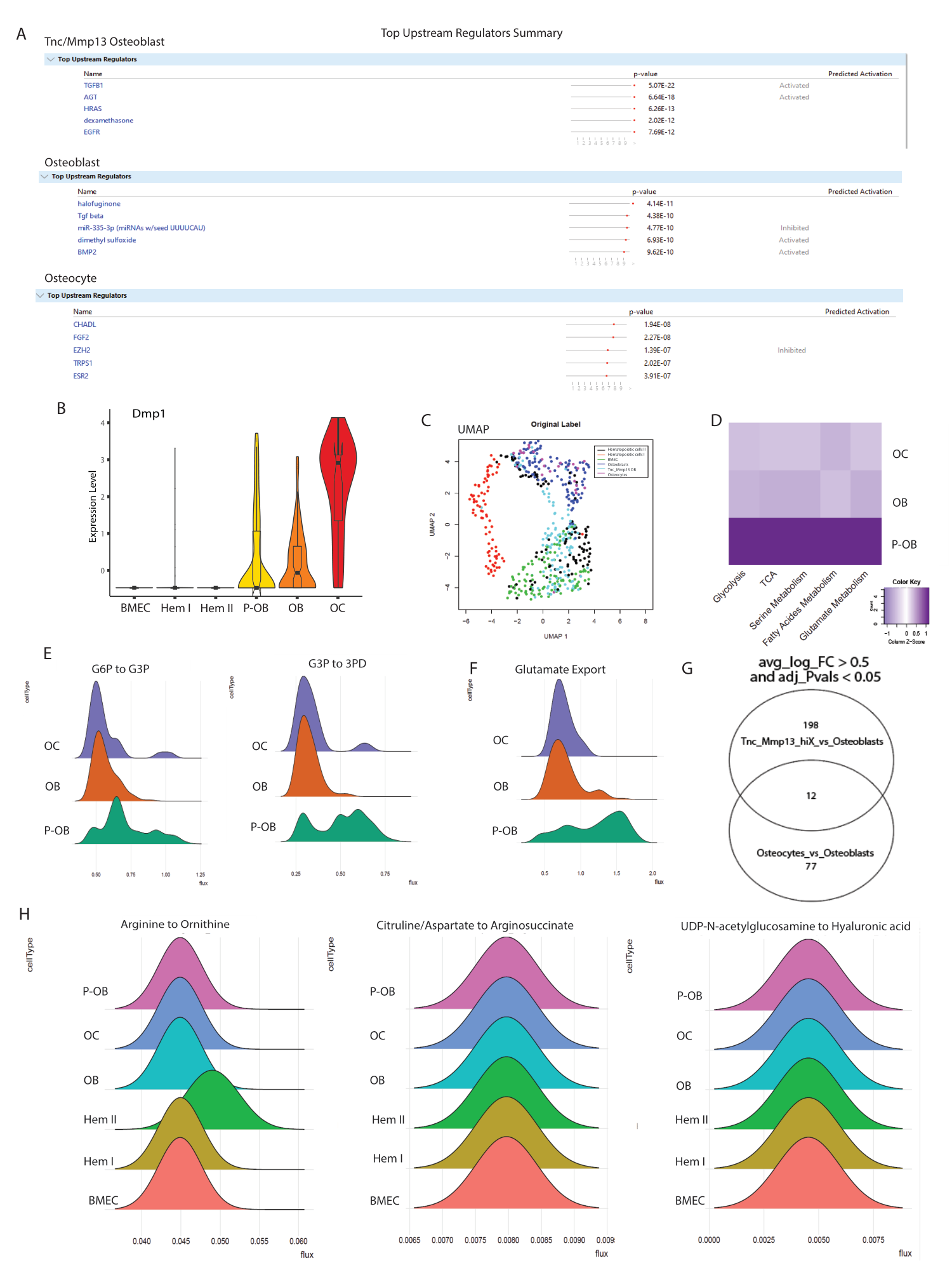

Supplement: Supplementary Figure 2 — (A). Predictive upstream regulators in Tnc/Mmp13 osteoblast (pre-osteoblasts), osteoblast and osteocyte. (B). Dmp1 expression in different cell types (C). scFEA UMAP. (D). Heatmap indicates the distribution of predicted cell-wise flux of glycolytic, TCA, serine metabolism, fatty acid metabolism and glutamate metabolism relative to pre-osteoblast (Tnc/Mmp13) values. The heatmap uses a column Z-score to show significant differences between pre-osteoblasts (Tnc/Mmp13), osteoblasts, and osteocytes. (E–F). Ridgeline plots indicate the distribution values of metabolic flux in pre-osteoblasts (P-OB), osteoblasts (OB), and osteocytes (OC). Each ridgeline represents the flux between two metabolites (x-axis) for different cells that are plotted on the y-axis. (G). Venn diagram shows the number of differentially expressed genes for Tnc_mmp13_osteoblasts vs osteoblasts, and osteocytes vs osteoblasts. H. Ridgeline plots indicate the distribution values of metabolic flux in pre-osteoblasts (Tnc/Mmp13), osteoblasts (OB), osteocytes (OC), hematopoietic cells (Hem I and Hem II), and bone marrow endothelial cells (BMEC). [file Image_2.tiff]

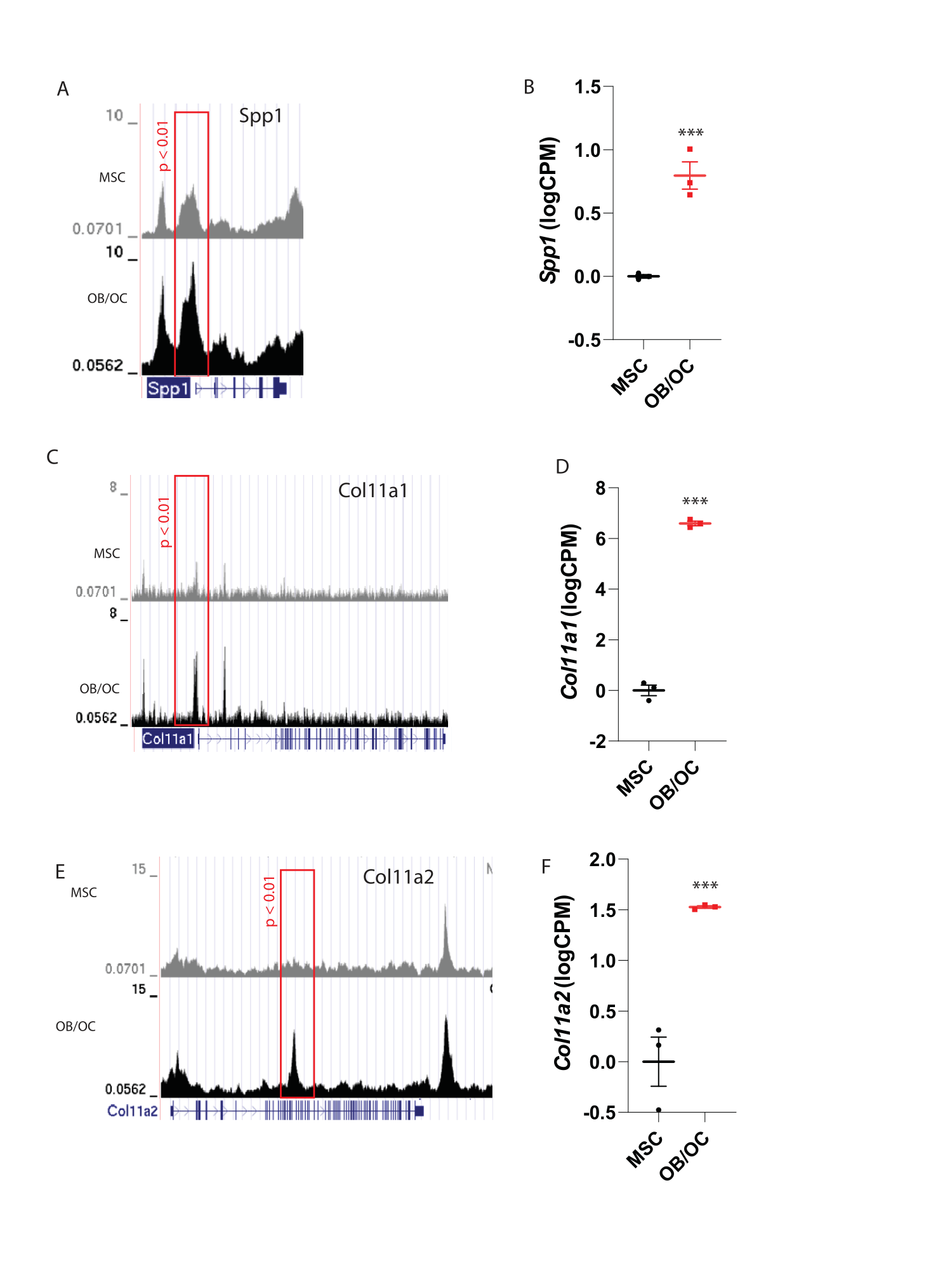

Supplement: Supplementary Figure 3 — (A–F). Chromatin accessibilities with corresponding gene expression of Spp1 (p = 0.00933495), Col11a1 (p = 1.9363E-05), and Col11a2 (p = 7.2591E-06) in differentiated (OB/OC) versus undifferentiated (MSC) cells. [file Image_3.tiff]

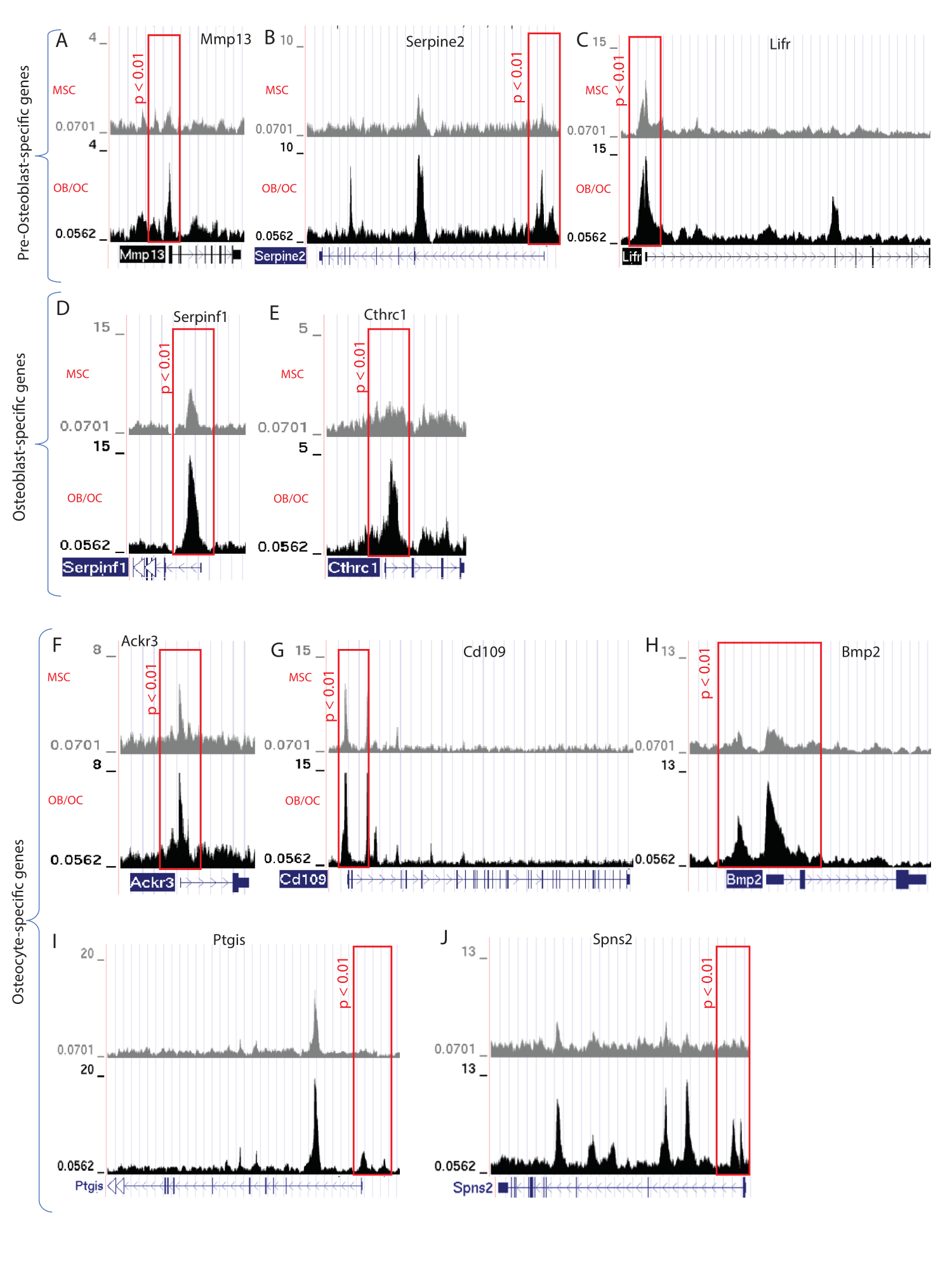

Supplement: Supplementary Figure 4 — (A–C). Assessment of chromatin accessibilities at Mmp13 (p = 0.01025011), Serpine2 (p = 3.4093E-09), and Lifr (p = 1.2547E-07) genomic regions. These genes were detected as highly enriched in pre-osteoblasts. (D–E). Assessment of chromatin accessibilities at Serpinf1 (p = 0.00099491), and Cthrc1 (p = 0.013) genomic loci. These genes were predicted to be highly enriched in osteoblasts. (D–E). Assessment of chromatin accessibilities at Ackr3 (p = 2.503E-06), Cd109 (p = 0.00179923), Ptgis (p = 6.8707E-06), Spns2 (p = 0.00521604), and Bmp2 (p = 0.00540629) genomic regions. These genes were detected primarily in osteocytes. Top tracks (gray) corresponded to undifferentiated cells (MSC) and lower tracks (black) corresponded to differentiated cells (OB/OC). [file Image_4.tiff]

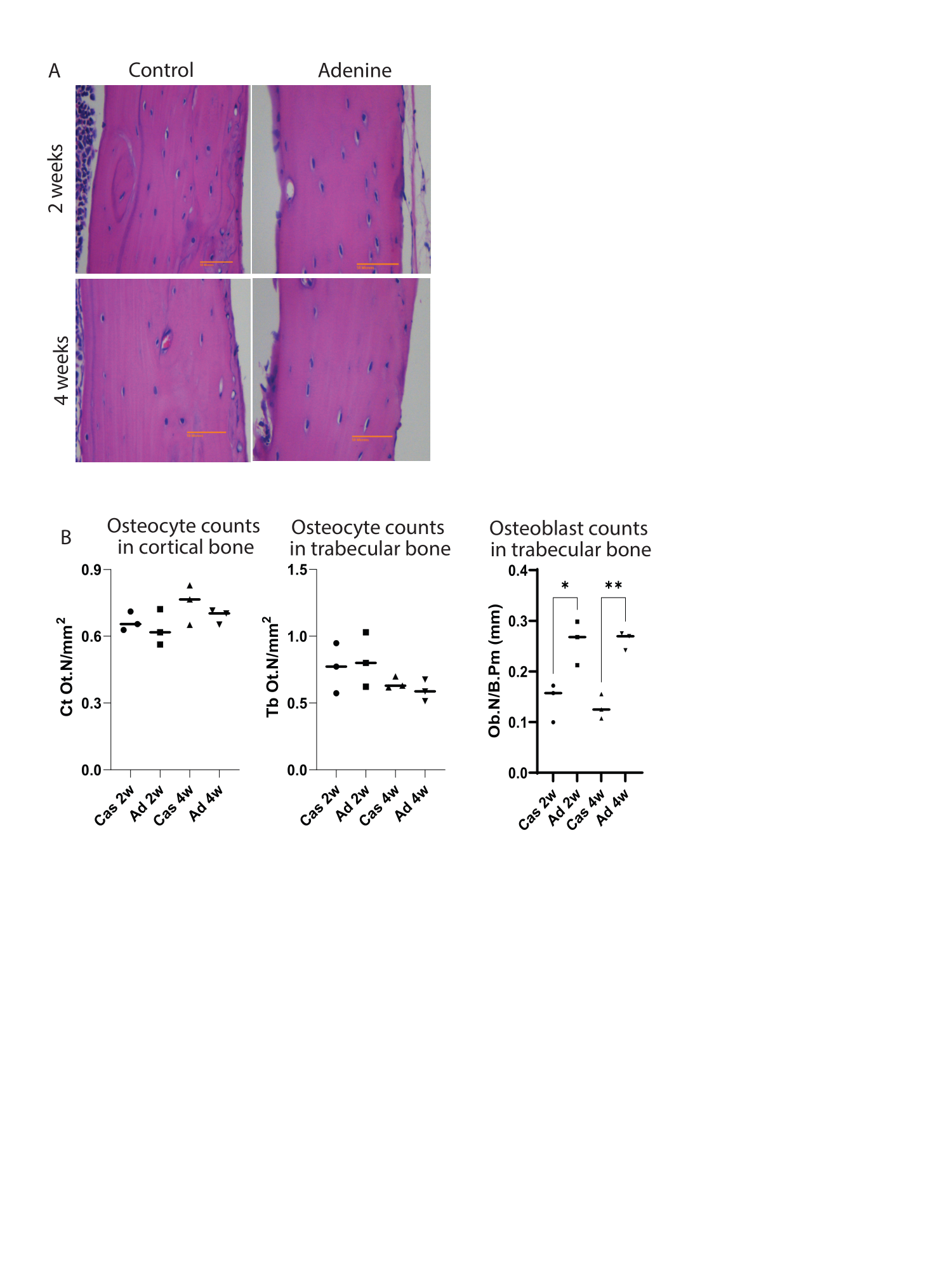

Supplement: Supplementary Figure 5 — Osteocyte and osteoblast cell numbers in cortical and trabecular bone. (A). The image shows osteocytes in the lacunae from femurs that were stained with hematoxylin and eosin. (B). Osteocyte numbers from cortical bone were counted in the midshaft of bone and normalized to bone area. For counting of osteocytes in trabecular bone, cells were counted in distal femur excluding endocortical surfaces and primary spongiosa, then normalized to trabecular bone area. Osteoblasts were counted in distal femur in the same trabecular region as where osteocytes were counted and normalized to trabecular bone surface. [file Image_5.tiff]
